# The Principles of SARS-CoV-2 Intervariant Competition Are Exemplified in the Pre-Omicron Era of the Colombian Epidemic

**DOI:** 10.1128/spectrum.05346-22

**Published:** 2023-05-16

**Authors:** Gregory S. Orf, Lester J. Pérez, Karl Ciuoderis, Andrés Cardona, Simón Villegas, Juan P. Hernández-Ortiz, Guy Baele, Aurash Mohaimani, Jorge E. Osorio, Michael G. Berg, Gavin A. Cloherty

**Affiliations:** a Infectious Disease Research, Abbott Diagnostics Division, Abbott Laboratories, Abbott Park, Illinois, USA; b Abbott Pandemic Defense Coalition (APDC), Abbott Park, Illinois, USA; c UW-GHI One Health Colombia, Universidad Nacional de Colombia Sede en Medellín, Medellín, Colombia; d Department of Microbiology, Immunology and Transplantation, Laboratory of Clinical and Evolutionary Virology, Rega Institute, KU Leuven, Leuven, Belgium; e UW-GHI One Health Colombia, University of Wisconsin—Madison, Madison, Wisconsin, USA; Emory University School of Medicine

**Keywords:** SARS-CoV-2, viral surveillance, Bayesian inference, phylogeography, Colombia, variant of interest, SIR model

## Abstract

The first 18 months of severe acute respiratory syndrome coronavirus 2 (SARS-CoV-2) infections in Colombia were characterized by three epidemic waves. During the third wave, from March through August 2021, intervariant competition resulted in Mu replacing Alpha and Gamma. We employed Bayesian phylodynamic inference and epidemiological modeling to characterize the variants in the country during this period of competition. Phylogeographic analysis indicated that Mu did not emerge in Colombia but acquired increased fitness there through local transmission and diversification, contributing to its export to North America and Europe. Despite not having the highest transmissibility, Mu’s genetic composition and ability to evade preexisting immunity facilitated its domination of the Colombian epidemic landscape. Our results support previous modeling studies demonstrating that both intrinsic factors (transmissibility and genetic diversity) and extrinsic factors (time of introduction and acquired immunity) influence the outcome of intervariant competition. This analysis will help set practical expectations about the inevitable emergences of new variants and their trajectories.

**IMPORTANCE** Before the appearance of the Omicron variant in late 2021, numerous SARS-CoV-2 variants emerged, were established, and declined, often with different outcomes in different geographic areas. In this study, we considered the trajectory of the Mu variant, which only successfully dominated the epidemic landscape of a single country: Colombia. We demonstrate that Mu competed successfully there due to its early and opportune introduction time in late 2020, combined with its ability to evade immunity granted by prior infection or the first generation of vaccines. Mu likely did not effectively spread outside of Colombia because other immune-evading variants, such as Delta, had arrived in those locales and established themselves first. On the other hand, Mu’s early spread within Colombia may have prevented the successful establishment of Delta there. Our analysis highlights the geographic heterogeneity of early SARS-CoV-2 variant spread and helps to reframe the expectations for the competition behaviors of future variants.

## INTRODUCTION

As SARS-CoV-2, the causative agent of COVID-19, trends toward an endemic status, a better understanding of its interepidemic molecular evolution will be required to inform public health responses and vaccination programs (1). While variant of concern (VOC) Omicron has dominated the pandemic landscape during 2022, it was preceded in time by a long list of other variants that appeared, briefly predominated, and then receded (2). These pre-Omicron variants competed with different outcomes across different countries and susceptible populations, leading to heterogeneous geographic spread (3–6).

While the emergence and invasion of the early major VOCs Alpha (Pango lineage B.1.1.7), Beta (Pango lineage B.1.351), and Gamma (Pango lineage P.1) have been well documented (7–9), these were the first variants of concern with significantly increased transmissibility compared to that of the original Wuhan strain. The subsequent phase of variant competition, in which one or more of these incumbents was replaced by a new one, especially after the start of vaccination efforts, presents additional complexities. One such situation existed in Colombia during the country’s third epidemic wave in early 2021, i.e., the Alpha and Gamma variants briefly became established before being completely replaced by variant of interest (VOI) Mu (Pango lineage B.1.621) (10). This same trajectory occurred in nearby Ecuador and Bolivia (https://outbreak.info/, accessed on 5 December 2022); however, their limited genomic surveillance precludes in-depth analysis.

At the beginning of the COVID-19 pandemic, the Colombian National Institute of Health (INS) created SARS-CoV-2 diagnostic and genomic surveillance networks to expand the testing and sequencing volumes, respectively. Through August 2022, the two networks had performed approximately 700,000 molecular diagnoses per million inhabitants and reported more than 22,000 genomic sequences to GISAID, placing Colombia among the top five countries in the Latin American and Caribbean (LAC) region in those metrics (11, 12). In this study, we utilized all SARS-CoV-2 sequences available from Colombia prior to September 2021 to conduct a molecular epidemiology study with a focus on pre-Omicron variant competition. We consider the variables of genetic diversity, transmissibility, and environmental factors using Bayesian phylodynamic analysis to understand the transmission behavior of the sequenced strains. Using a susceptible-infectious-recovered (SIR) epidemiological model, we explore public health mitigations (e.g., vaccinations) and immune evasion behavior to delve further into how these variants competed within the susceptible population. We then discuss how the principles governing Mu’s trajectory of emergence, displacement, and establishment can be considered when making predictions about future variants. Future interepidemic events of SARS-CoV-2 will be shaped by intervariant competition, driven by antigenic drift in response to acquired immunity in the human population or independent spillovers.

## RESULTS

### The Colombian pandemic during early to mid-2021 was defined by competition between variants and the first administration of vaccines.

**FIG 1:**
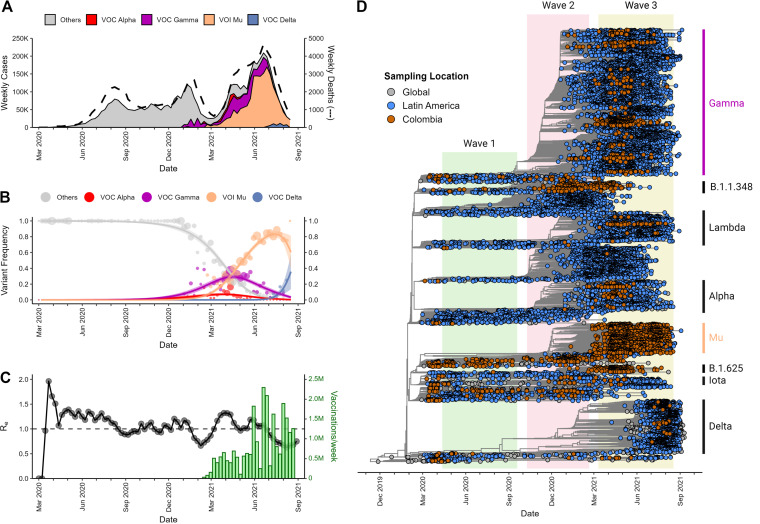
National-level lineage diversity during the first three local SARS-CoV-2 epidemic waves in Colombia. (A) Weekly cases of SARS-CoV-2 variants, estimated from the number of positive tests, and variant frequencies ascertained through sequencing (stack plot) overlaid with the weekly death count irrespective of variant. (B) Variant frequencies over time; the sizes of the points are proportional to the numbers of sequences collected per week, and the points are fitted with a logistic regression (shading represents the 95% confidence interval). (C) Estimation of effective reproduction numbers (*R_e_*s) of the virus over time estimated from case count alone (black points and line) and compared to vaccination efforts (green bars). (D) Time-resolved maximum-clade-credibility (MCC) tree estimated from 17,036 full genomes containing a global representation (gray dots), all high-quality data from Latin America (blue dots), and all high-quality data available from Colombia (orange dots). Clades formed by variants of concern or interest are labeled.

In the first 18 months after the appearance of SARS-CoV-2 in Colombia, the local epidemic was characterized by three major groupings of cases: wave 1 from May 2020 to September 2020, wave 2 from October 2020 to February 2021, and wave 3 from March 2021 to August 2021 (Fig. 1A). In January 2021, the first cases linked to a VOC appeared: Gamma constituted a relatively low number of cases near the end of wave 2. As wave 3 began in March 2021, there was an evident cocirculation of non-VOC strains, Gamma, Alpha, and Mu (Fig. 1A and B).

Available case count data enabled estimation of the effective reproduction number (*R_e_*) of the virus, which can describe the expansion of the epidemic (Fig. 1C). Increases in *R_e_* above 1 reflect the beginning of each wave. *R_e_* remained above 1 from roughly March 2020 until August 2020 and then temporarily decreased below 1 before rising again to just above 1 from October 2020 to June 2021, coinciding with wave 2. *R_e_* rose to roughly 1.5 again by March 2021, coinciding with the beginning of wave 3. By this time, mass vaccinations had begun in Colombia (Fig. S1 in the supplemental material), eventually reaching about two million vaccinations per week by June 2021 (Fig. 1C, green bars).

To characterize the genetic composition of SARS-CoV-2 circulating in Colombia within the greater context of the LAC region and global pandemic, we estimated a time-calibrated maximum-clade-credibility (MCC) tree using BEAST v.1.10.5pre_thorney_v0.1.1 (https://beast.community/thorney_beast) (Fig. 1D). A total of 17,036 sequences were evaluated, including 2,867 from Colombia (orange). The Colombian sequencing efforts and evaluation of potential biases in data set construction are shown in Fig. S2, S3, and S4. Consistent with the variant frequencies (Fig. 1A and B), wave 1 was constrained to only a few lineages basal to VOCs and VOIs. As discussed above, wave 2 saw the arrival of Gamma and Mu, though only at its end; much of wave 2 was characterized by lineages B.1.111 and B.1.1.348. Though these lineages were common at the time in the LAC region, they did not contain any of the hallmark spike mutations found in VOCs or VOIs (Table S1).

During the initial stages of wave 3 (March to April 2021), numerous sublineages within Gamma and Mu were represented within Colombia. As wave 3 progressed (May to July 2021), most of the sublineages within Gamma disappeared from Colombia while persisting in other LAC countries, such as neighboring Brazil. Conversely, substantial diversity persisted for Mu in Colombia. Additionally, the relatively few Colombian sequences belonging to VOC Alpha and VOI Lambda mostly formed monophyletic groups, indicating very few introductions of these variants into the country.

**FIG 2:**
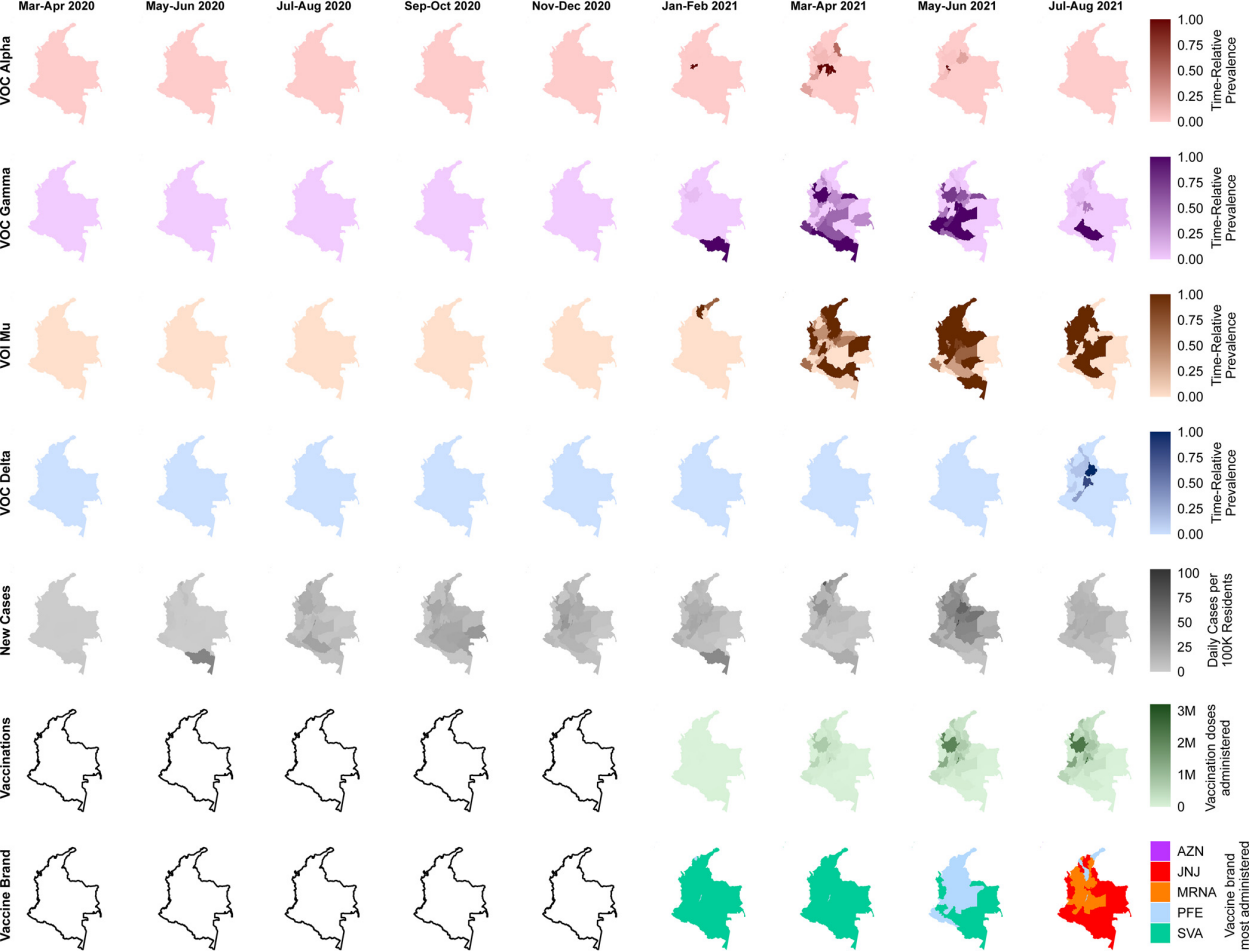
Subnational dynamics of the first three local epidemic waves of SARS-CoV-2 in Colombia. Spatiotemporal representations of various epidemiological and genetic factors are broken down at the department level, including time-relative prevalences of variants of interest or concern, daily cases per 100,000 residents, vaccination doses administered, and most-administered vaccine brand. For the variants, the scales represent the prevalence of each at a given time divided by its own maximum over the entire study period. Vector data used for producing maps are public domain and were obtained from Natural Earth (naturalearthdata.com; accessed on 17 February 2022).

We developed a detailed picture of the spatiotemporal evolution of the pandemic at the subnational level to better understand the factors influencing these patterns (Fig. 2 and Fig. S5). As wave 2 waned and wave 3 began, the ingress of VOCs and VOIs began. The appearance of Alpha began in Caldas, in the center of the country, before spreading in a limited fashion to surrounding departments in and north of the Andes Mountains (Fig. 2, top row). Gamma first appeared in Amazonas, along the southern border with Brazil, before spreading northward across the country (Fig. 2, second row). Mu, on the other hand, first appeared in Magdalena and La Guajira, in the north of the country near the border with Venezuela (Fig. 2, third row). By the time Mu supplanted Gamma in terms of total number of infections, Mu remained dispersed country-wide, while Gamma was restricted again to the south. Finally, near the end of wave 3, VOC Delta appeared in the major population centers in the center of the country, including Bogotá, D.C., Cundinamarca, and Santander (Fig. 2, fourth row). Delta, however, did not become a major driver of cases within the country, as overall case counts remained low until the import of Omicron in late 2021 (https://ourworldindata.org/coronavirus, accessed on 7 February 2022; beyond the temporal scope of this study).

Vaccination efforts began across the country in late February 2021, concurrent with the appearances of VOCs and VOIs (Fig. 2, fifth and sixth rows). Initial deliveries through unilateral negotiations by the Minister of Health and Social Protection provided primarily the Pfizer-BioNTech vaccine for the populous Bogotá, D.C., and the Sinovac vaccine throughout the rest of the country (Fig. 2, bottom row). By May to June 2021, the Pfizer-BioNTech vaccine became more widely used until the arrival of the Janssen and Moderna vaccines, which became prevalent by August 2021. Total vaccination efforts were spread across the 33 departments of Colombia based on population count, rather than COVID-19 case load. Overall, by the end of the study period in August 2021, roughly 30 million doses of vaccines had been administered; by that point, all age, employment, and comorbidity groups were eligible for vaccination. Notably, the areas which received the largest share of vaccinations (i.e., the most populous, primarily in and north of the Andes Mountains) (Fig. S6) were most affected by Mu rather than Gamma during the peak of wave 3 in May to June 2021.

### Genetic diversity and transmissibility of locally diversifying lineages.

**FIG 3:**
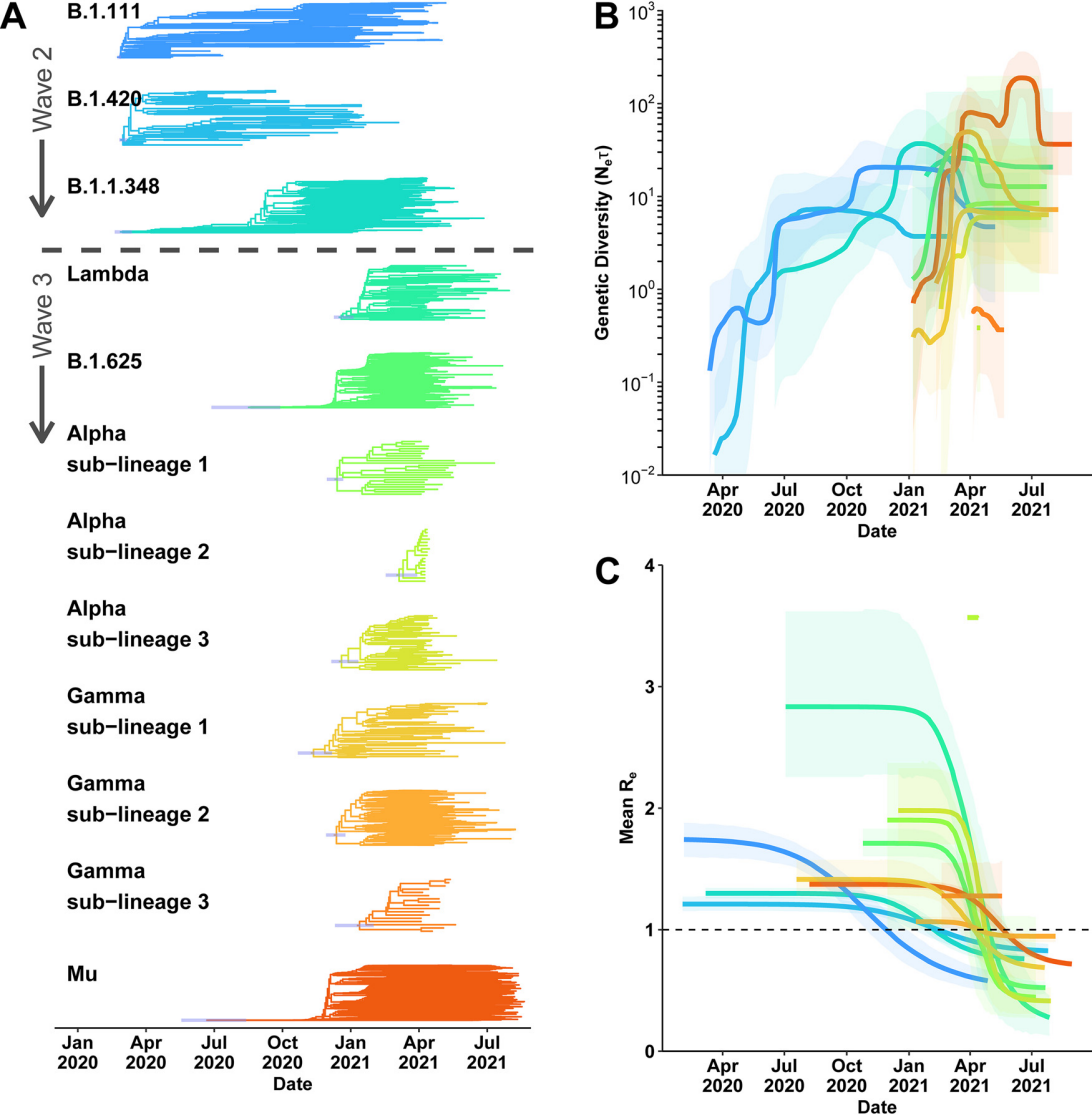
Evolutionary history, demographic reconstruction, and effective rates of reproduction of the major SARS-CoV-2 lineages circulating in Colombia during the study duration. (A) Clades extracted from the time-resolved maximum-clade-credibility (MCC) tree presented in Fig. 1 for the 12 SARS-CoV-2 lineages of interest. The 95% highest posterior density (HPD) interval for the ancestral node of each clade is indicated with a blue bar (time to most recent common ancestor [TMRCA] and confidence intervals are included in the supplemental material). (B) A demographic history of only the Colombian sequences from each of the lineages inferred via a Bayesian skyline plot (BSP) with coalescent tree prior and an exponential, uncorrelated clock model. The lines are the medians of the inferred products of effective population size and generation time (*N_e_τ*), and the shading represents the 95% HPD. (C) The reconstructed effective reproduction rates (*R_e_*s), computed from the posterior birth-death rates and SIR trajectories, deconvolved for only the Colombian sequences from each of the lineages. The lines are the median *R_e_* profiles, and the shading represents the 95% HPD.

To understand which features of Mu resulted in its greater prevalence, we first analyzed its genetic diversity and transmissibility characteristics in comparison with those of other viral lineages in the country (Fig. 3). From the MCC tree shown in Fig. 1D, we identified 12 main monophyletic clades with evidence of local diversification in Colombia (Fig. 3A and Fig. S7). We extracted the Colombian sequences and performed Bayesian skyline reconstruction (Fig. 3B) and effective reproductive rate estimation using the birth-death susceptible-infectious-recovered (BDSIR) model (Fig. 3C and Fig. S8) on each clade.

The main locally diversifying lineages contributing to waves 1 and 2, such as B.1.111, B.1.420, and B.1.1.348, were introduced into the country around the same time early in 2020 (Fig. 3A and Table S2). There were relatively equal degrees of genetic diversity within these three lineages (Fig. 3B) until November 2020, after which it was increased in B.1.1.348 until the arrival of the VOCs and VOIs in January 2021. Regarding transmissibility, B.1.111 had the highest *R_e_* value from March 2020 until October 2020, after which B.1.420 and B.1.1.348 maintained the highest *R_e_* values until March 2021. These characteristics resulted in B.1.111 maintaining at least a 25% share of the total cases for the entire first year of the local epidemic (March 2020 until March 2021) (Fig. S5).

In contrast, the main lineages contributing to wave 3 were introduced into the country at different times: Gamma and Lambda were introduced first between July and October 2020, followed by Alpha, B.1.625, and Mu between October 2020 and January 2021 (Fig. 3A and Table S2). After Lambda’s importation, despite its high level of genetic diversity and transmissibility (Fig. 3B and C), it failed to further diversify and establish large-scale transmissions outside Valle del Cauca, where the vaccination rate was low (Fig. 2 and Fig. S5). In contrast, the other lineages increased in genetic diversity for approximately 3 months, after which they declined, except for Mu, which reversed its decline and trended upward until peaking in June 2021 (Fig. 3B). This inflection could be indicative of a bottleneck effect that favored the further diversification of this lineage. In addition, we observed that Mu’s spike in genetic diversity between May and June 2021 appeared concomitantly with a maintenance of *R_e_* above 1 (Fig. 3C), while other variants had already declined in both measures (Table S3). These observed advantages could have been driven by a variety of factors both extrinsic (e.g., environmental, interventional, or demographic) and intrinsic (e.g., viral fitness).

### Continuous phylogeographic reconstruction reveals environmental factors influencing transmission dynamics of SARS-CoV-2 in Colombia.

**FIG 4:**
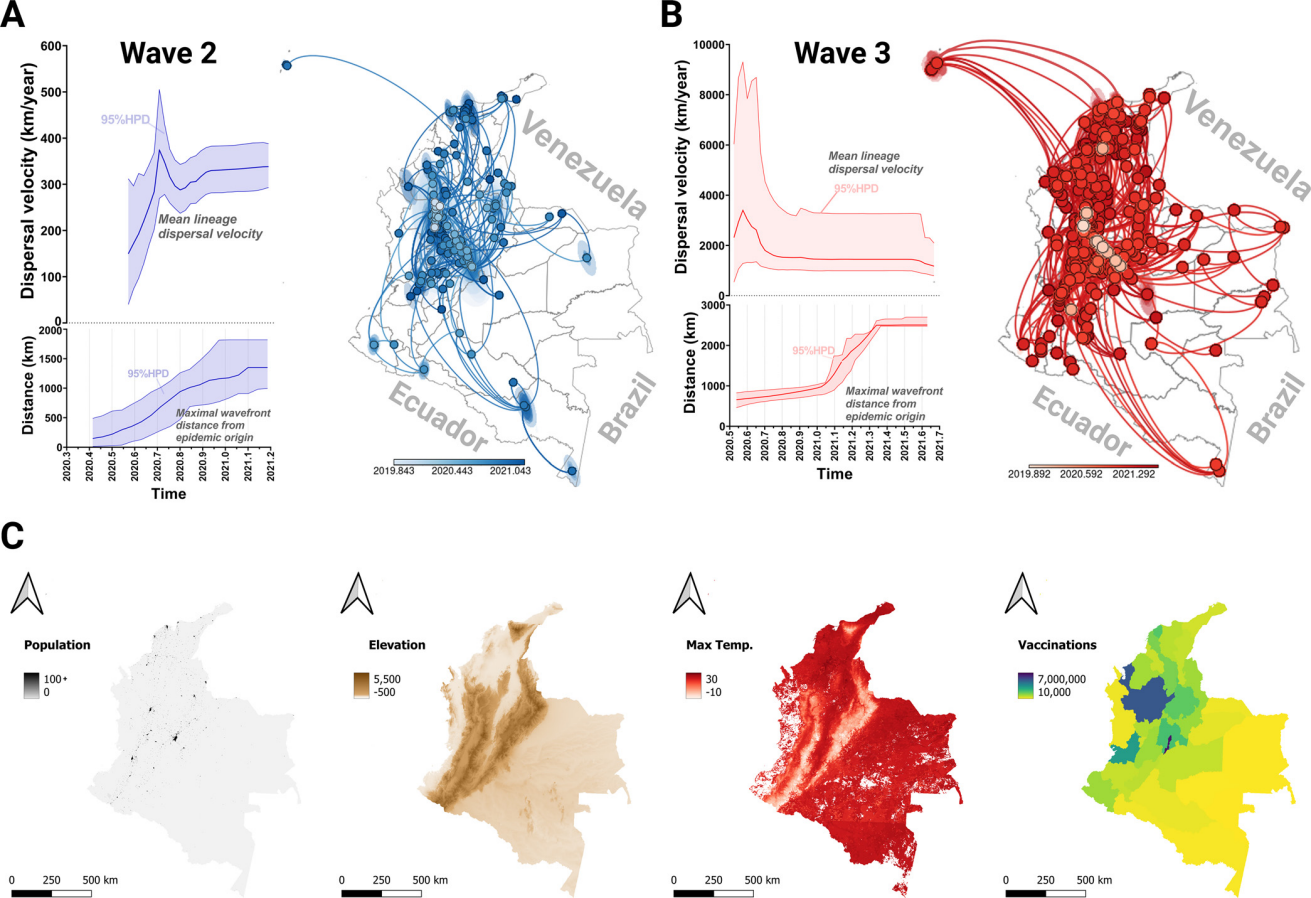
Local spatiotemporal diffusion of SARS-CoV-2 across Colombia computed with continuous phylogeographic reconstruction. (A and B) Geographic spread of SARS-CoV-2 in Colombia during waves 2 (A) and 3 (B). The sizes of the shaded polygons surrounding the solid circles are proportional to the numbers of MCC tree lineages that diffused in the respective geographic locations upon arrival, and the color relates to the time at which the lineage first reached the location. Dispersal statistics are also reported, including changes in the mean lineage dispersal velocity over time and the evolution of the maximal wavefront distance from the origin of the epidemic (95% HPD regions are shaded). (C) Environmental variables tested for their impact on the dispersal velocity (resistance and conductance) and distance (attraction and repulsion). See Tables S4 to S6 for the results from each environmental raster. Vector data used for producing maps are public domain and were obtained from Natural Earth (naturalearthdata.com; accessed on 17 February 2022).

Continuous phylogeographic inferences based on a Bayesian framework can summarize the dispersal history of viral dynamics by exploring the Colombian monophyletic clades and some of the extrinsic factors mentioned above (13). The first wave of the local epidemic of SARS-CoV-2 infections in Colombia was characterized by the lack of connections between the strains present, indicating that this wave resulted mainly from introductions of foreign lineages (BEAST XML and log files are available; see “Data availability” below). Therefore, we focused our local phylogeographic reconstruction on the monophyletic clades specific to waves 2 and 3 (Fig. 4). The lineages involved in wave 2 were dispersed across the entire country with significant local diffusion events (Fig. 4A). In contrast, the lineages involved in wave 3 appear to be associated with local diffusion in mostly the northwest and central regions (Fig. 4B), where most of the population resides (Fig. S6).

From the information embedded in the inferred phylogenies, we determined dispersal statistics, including the branch dispersal velocities and diffusion coefficients (Fig. 4A and B, left) as described by Dellicour et al. (14). We observed that the lineages of wave 3 (primarily Gamma and Mu) dispersed at a much higher weighted dispersal velocity (~1,654 km/year) than those involved in wave 2 (primarily B.1.111 and B.1.1.348; ~323 km/year). However, there was no significant difference in the extent of spatial dissemination between the two waves, with maximum wavefront distances between 1,800 and 2,500 km, essentially spanning the country.

Finally, we investigated the potential role of various environmental variables in the dispersal direction and heterogeneity of dispersal velocities observed between both waves. For this, we used the analytical framework implemented in the R package SERAPHIM (15). In all cases, we used path models to test the influence of environmental variables falling under demographic (population density and levels of vaccination), geographic (elevation), and climatic (daily maximum temperature) classifications (Fig. 4C). We found strong support for two variables impacting the directional spread of lineages: daily maximum temperature considered as a resistance factor during wave 2 (i.e., local diffusion was lowest through regions with higher temperatures), and the geographic elevation considered as an attraction factor during wave 3 (i.e., local diffusion was higher through higher-elevation regions) (Table S4). These results seem sufficient to explain the observation that there was less local diffusion in wave 3 than in wave 2 (Fig. 4A and B, shaded polygons).

In the case of the dispersal velocity, none of the environmental factors tested led to a Q distribution higher than 90% positive values (Table S5). This result was consistent despite perturbing the scale of the rasters (Table S6) (16), suggesting that the differences in dispersal velocities of the lineages of both waves did not depend on the environmental factors tested, but might instead have depended on intrinsic characteristics of those lineages.

### Mu is predicted to evade prior immunity imparted by infection with non-VOCs, Alpha, and Gamma.

We then focused on intrinsic factors that may have contributed to Mu’s dominance in Colombia. Logistic growth and marginal means methods within a susceptible-infectious-recovered (SIR) framework (17, 18) can be used to calculate the growth advantage (ρ) of one lineage (or group of lineages) over another. The growth advantage (ρ) estimated in the applied model is linked to various intrinsic factors, including an increase in transmissibility (τ, which may also be linked to an increase in peak viral load), increase in infectious duration (κ), enhanced immune evasion behavior (ε, which can vary from complete cross-protection, ε = 0, to full evasion, ε = 1, as defined previously [17]), or some combination of all. As previously assumed (18, 19), we consider κ to be identical between all lineages in question due to numerous studies citing the inability to culture replication-competent virus from patient specimens obtained after 10 days post-symptom onset (20–24). Thus, an interplay between τ and ε will affect ρ. Additionally, if ε does indeed play a role, its contribution strongly depends on the assumed level of population seroprevalence (Ω) against the virus through previous infection or vaccination.

**FIG 5:**
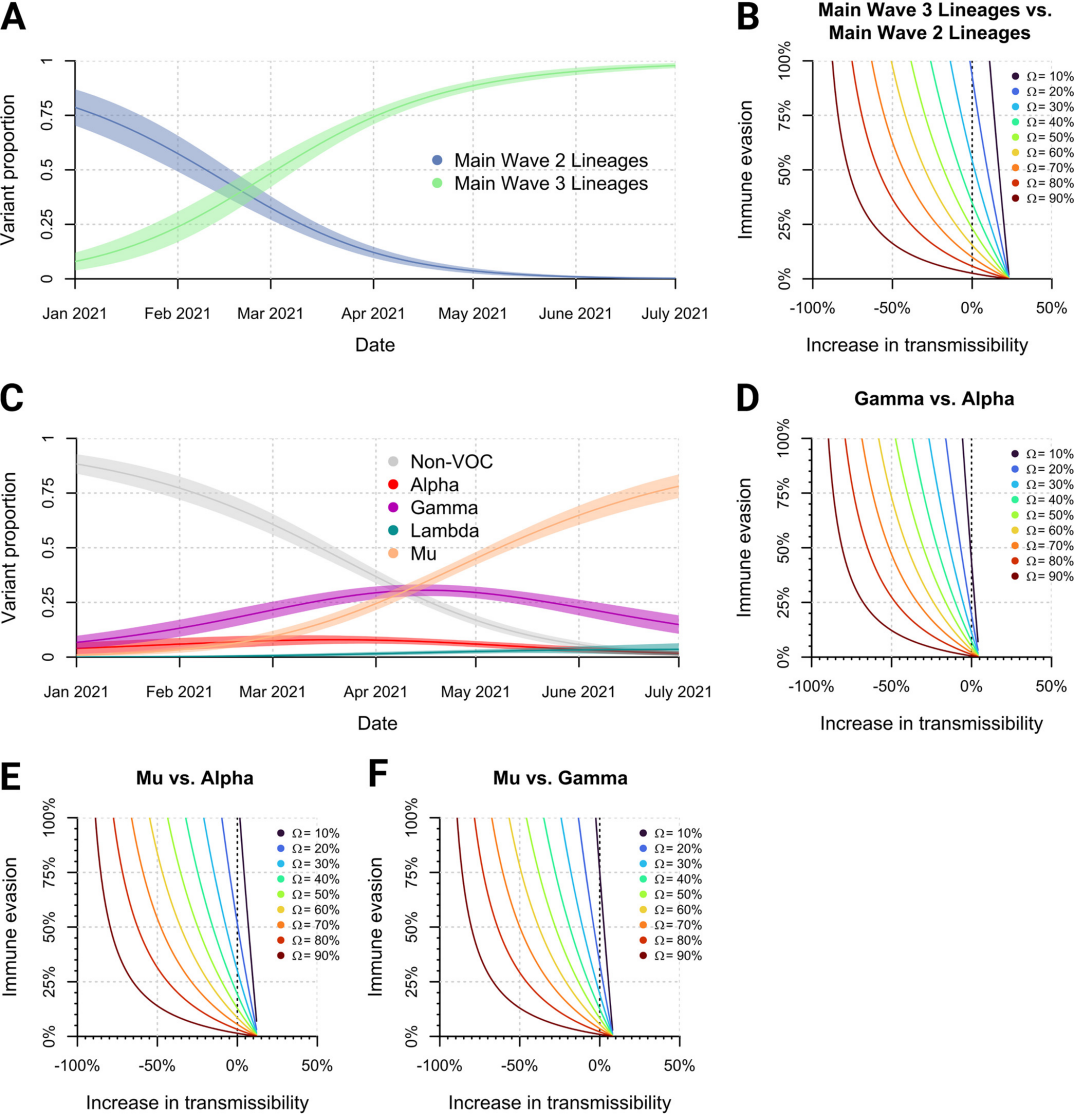
The growth of variants in Colombia between March and July 2021 and the relationship between their potential increases in transmissibility and immune evasion characteristics. (A) The period between January and April 2021 was marked by the transition between wave 2 and wave 3 and the composite variant lineages within. (B) The level of population seroprevalence (Ω) against the wave 2 (and earlier) lineages strongly influences the relationship between increases in transmissibility and immune evasion characteristics for the collective wave 3 lineages entering the population. (C) The period between March and June 2021 was marked by a competition between variants Alpha, Gamma, and Mu, with Mu dominating from May 2021 until the end of the third local epidemic wave. (D, E, and F) The transmissibility-immune evasion-seroprevalence relationships for new Gamma infections introduced against a backdrop of immunity against previous Alpha infections (D), new Mu infections introduced against a backdrop of immunity against previous Alpha infections (E), and new Mu infections introduced against a backdrop of immunity against previous Gamma infections (F).

We first applied this model to understand how the lineages mainly composing wave 2 (B.1, B.1.111, B.1.420, and B.1.1.348) competed against those mainly composing wave 3 (Alpha, Gamma, Mu, and B.1.625). This estimation (Fig. 5A) revealed that the wave 3 lineages collectively had a growth advantage of 4.6% per day (95% confidence interval [CI], 3.8 to 5.4%) over the wave 2 lineages. An analysis of this interplay (Fig. 5B) shows that, if immune evasion behavior were completely absent (and thus seroprevalence played no role), the main wave 3 strains would be ~25% more transmissible than the wave 2 strains. Data collected by the INS indicate a seroprevalence level (Ω) of ~20 to 30% averaged across the country in March to April 2021 (https://www.ins.gov.co/estudio-nacional-de-seroprevalencia/reporte.html#curso, accessed on 11 August 2022). Thus, even with a modest increase in transmissibility, immune evasion of 25 to 50% in wave 3 lineages would be necessary to satisfy their calculated growth advantages over the wave 2 lineages.

To understand the contributions of Alpha, Gamma, Lambda, and Mu within wave 3, we performed the same analysis, inferring the growth advantages of each variant versus the others. The logistic growth model (Fig. 5C) revealed that in the period of March to June 2021, Gamma had a growth advantage over Alpha of 0.8% per day (95% CI, −0.1 to 1.8%), Mu had a growth advantage over Alpha of 2.8% per day (95% CI, 1.8 to 3.8%), and Mu had a growth advantage over Gamma of 1.9% per day (95% CI, 1.3 to 2.6%). Alpha, Gamma, and Mu had growth advantages over non-VOC strains of 1.5% (95% CI, 0.5 to 2.4%), 2.3% (95% CI, 1.7 to 2.9%), and 4.2% (95% CI, 3.5 to 5.0%) per day, respectively. Lambda never rose high enough in prevalence to allow calculation of growth advantage due to its failure to further diversify and spread once in Colombia.

When we consider the relationships between ε, τ, and Ω that can explain the calculated growth advantages of Alpha, Gamma, and Mu (Fig. 5D to F), we observe that, if immune evasion behavior were completely absent (i.e., ε = 0), Gamma would be 5% more transmissible than Alpha, Mu would be 15% more transmissible than Alpha, and Mu would be 10% more transmissible than Gamma. In this same scenario, Alpha, Gamma, and Mu would be ~8%, ~13%, and ~20% more transmissible, respectively, than non-VOC strains (Fig. S9). Mu has been noted to evade neutralizing antibodies against the B.1 (non-VOC) lineage in the serum of vaccinated individuals at rates of roughly 60 to 80% (10). This level of immune evasion is achieved in our model (Fig. S9) at 20% seroprevalence without any required increase in transmissibility, confirming that Mu would prevail over other non-VOC strains.

The level of seroprevalence at the time of maximal variant competition (20 to 30%) dictates that for Gamma to overcome Alpha, Gamma would need either a combination of a <5% increase in transmissibility plus 10 to 20% immune evasion or a decrease in transmissibility plus higher levels of immune evasion (Fig. 5D). For Mu to overcome Alpha at the same seroprevalence, there is a higher level of flexibility within the model to accommodate a combination of a >5% increase in transmissibility plus 25 to 50% immune evasion (Fig. 5E). For Mu to overcome Gamma at the same seroprevalence, assuming a 5% increase in transmissibility, 15 to 25% immune evasion would be required to justify the observed growth advantage (Fig. 5F). Mu’s ascendancy over Gamma directly, according to these models, is consistent with a modest (<10%) increase in transmissibility coupled to a modest (<20%) degree of immune evasion.

### Most of the international spread of VOI Mu was driven by export from the United States.

**FIG 6:**
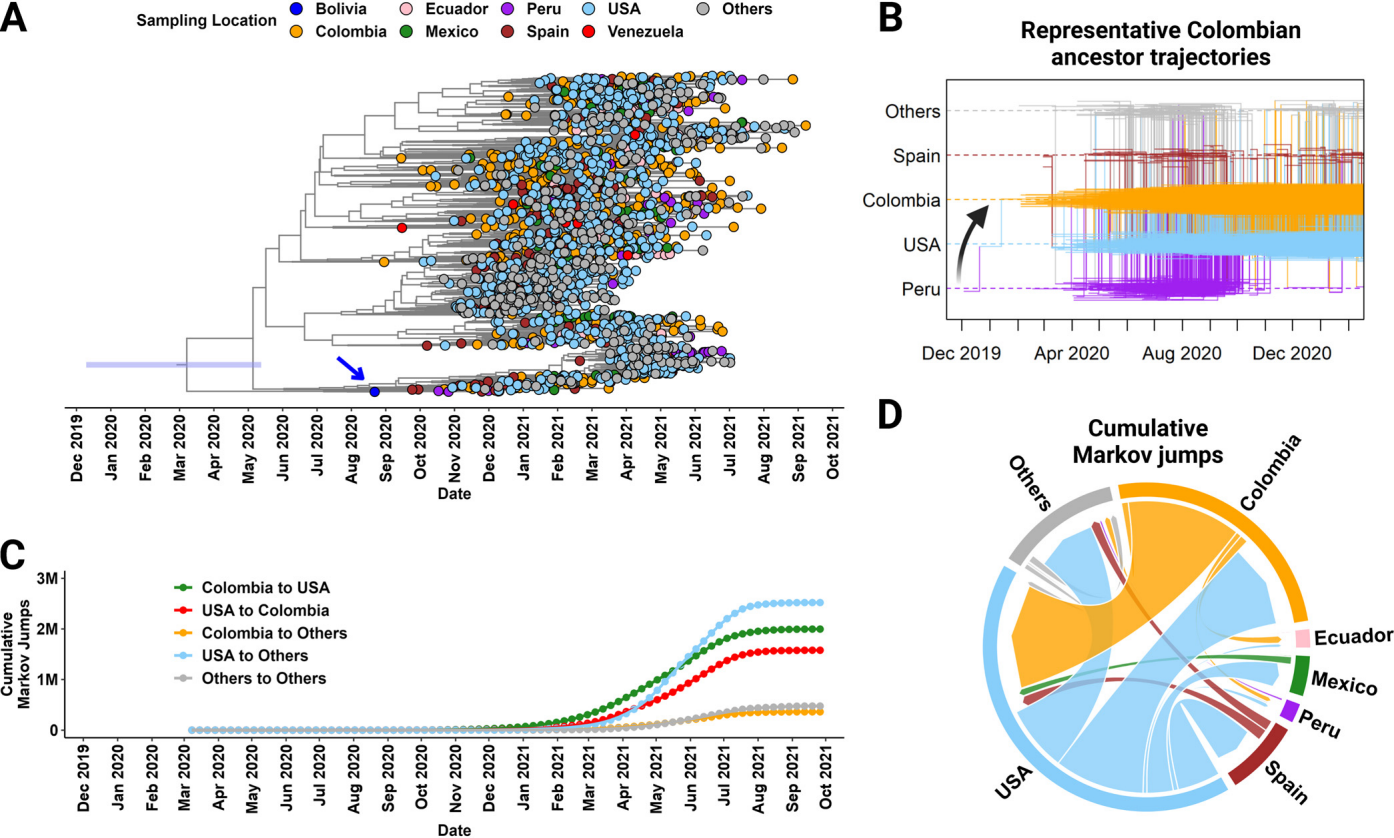
Discrete-state phylogeographic reconstruction of the appearance and global dissemination of the Mu variant. (A) Time-resolved MCC tree estimated from a curated set of 1,833 sequences collected worldwide. The colors of the tips represent the sampling locations indicated in the key. The 95% HPD interval for the root is indicated with a blue bar. The arrow denotes the earliest sequence identified, which was collected in Bolivia. (B) Plot of Markov jump trajectories representative of six foundational sequences across the MCC tree shown in panel A. The horizontal lines represent times when a location state was maintained, while the vertical lines represent times when a location state jumped between two locations. (C) Time-dependent representation of the cumulative Markov jump trajectories calculated for the entire MCC tree. (D) Time-independent representation of migration flow derived from the cumulative Markov jump trajectories calculated for the entire MCC tree over the entire study period. The widths of the links indicate the relative frequencies of viral movements, and the direction of the migration flow is from the origin to the destination as denoted by the arrowhead-shaped end.

We utilized a discrete phylogeographic inference method incorporating known travel history (25) to reconstruct the early international transmission events that led to the establishment of Mu in different countries (Fig. 6). The MCC tree obtained from 1,828 world-wide representative Mu sequences indicated that the most recent common ancestor of this lineage emerged in June 2020 from Bolivia (Fig. 6A). We also identified the most ancestral Colombian strains from six of the major clades within the MCC tree and performed a Markov jump analysis, which describes the necessary events that precede viral emergence (Fig. S10). These plots consistently revealed an early contribution of Peru and the United States to these foundational Colombian strains, which arrived by February 2020 (a representative plot is shown in Fig. 6B).

We next summarized the Markov jumps inferred from the entire MCC tree to quantify the directionality and magnitude of the international spread of Mu (Fig. 6C). This revealed that a significant portion of the total international migration of Mu took place between Colombia and the United States, with spread in both directions. However, the United States was the major driver of migration to other countries, especially to Mexico and European countries, such as Spain. A time-dependent representation of these Markov jumps (Fig. 6D) shows that the major sharing of Mu between Colombia and the United States began in December 2020 to January 2021 and preceded the start of the United States’ export of Mu to other countries, which began in February to March 2021. Tertiary spread began increasing in April to May 2021, especially between Spain and other European countries, such as Italy and the Netherlands.

## DISCUSSION

The COVID-19 pandemic can currently be delineated into three stages: the prevariant period, the intervariant competition period, and the inter-Omicron competition period. Here, we focus on the mechanisms underlying the establishment and competition of the VOCs and VOIs during the first two stages, using Colombia as an example where this process unfolded. During the initial competition period, different variants thrived in different geographic locations: for example, whereas Alpha played a major role in Europe and Beta in southern Africa, these were not particularly impactful in other areas, such South America. With Gamma appearing and proliferating in neighboring Brazil, its presence in Colombia was limited by Mu. The factors that drove this outcome in Colombia are generalizable to our understanding of the evolutionary dynamics of SARS-CoV-2 in other geographic regions.

Numerous studies using modeled data or infection metadata have predicted the contributions of introduction time and immune evasion to the fitness advantages of variants as they increase in prevalence in communities (26–32). Recent studies on Delta and Omicron using genomic data (18, 33, 34) are in concordance with these models, but there are few studies addressing these aspects with other VOCs/VOIs during the pre-Omicron period. Sequences collected during the three initial epidemic waves in Colombia provided an opportunity to shed light on the transition from a landscape of multiple basal lineages to another shaped by the import and local diversification of just a few VOCs/VOIs. Our time-scaled phylogenetic results (Fig. 1), in concordance with other studies (6), indicate that the basal lineages that would eventually develop into VOCs/VOIs emerged early in the pandemic but did not spread efficiently among the backdrop of other lineages until they gained in fitness and encountered favorable environmental factors. Specifically, in Colombia, we observed that once vaccinations began, the major lineages of wave 2 (B.1, B.1.111, and B.1.1.348) declined and selective pressure favored effective transmission and diversification of the variants harboring the hallmark spike mutations that evade immunity (e.g., E484K [a change of E to K at position 484]), as well as influencing their geographic distribution (Fig. 2 and Table S1).

Over time, factors including interlineage competition and environmental variables influenced the increases in genetic diversity and transmissibility (i.e., effective reproduction number) of VOC/VOI lineages that outcompeted the basal lineages (Fig. 3). Furthermore, the VOCs/VOIs of wave 3 dispersed across the country with higher velocities than the basal lineages of wave 2, though elevation was a major environmental factor that restricted the extent of their geographic spread (Fig. 4). In Colombia, the Andes Mountains separate the coastal northwest from the tropical Amazon basin in the southeast, with most of the major population centers, such as Bogotá and Medellín, contained within the mountain range itself. Due to this, movement between distant areas is difficult and presumably hampers viral dispersal.

There was no clear evidence of a genetic diversity advantage among the wave 3 lineages until April 2021, when Mu’s genetic diversity spiked (Fig. 3B). This event was coincident with the widespread adoption of vaccinations (Fig. 2). In the most populous region, Bogotá, D.C., the Pfizer-BioNTech brand was the most administered vaccine from January 2021 to August 2021 and appeared to be effective in restricting the spread of all lineages by May 2021, except for Mu (Fig. 2). In fact, Mu was able to effectively spread across the country despite the growing vaccination rate and growing levels of infection-mediated immunity, indicating a high level of immune evasion against both. This is reflected in the Bayesian skyline plot (BSP) profiles (Fig. 3B), which illustrate a genetic bottleneck effect for all of the main wave 3 lineages except Mu, despite Mu not having the highest calculated level of transmissibility (Fig. 3C). Our logistic growth model (Fig. 5) confirmed that immune evasion characteristics alone, against either immunization or previous infection by any other local strain, could justify the observed growth advantages in Mu. This finding can explain how, after its importation into the country (Fig. 6), Mu became the predominant variant.

Growth advantages through increases in immune evasion characteristics (or transmissibility) in a variant are biochemically derived from the constellation of mutations present, which have been extensively characterized by other groups (Table S1). However, our analysis agrees that genetic profile alone does not determine the successful establishment of a variant in a region with other cocirculating lineages; indeed, multiple models (3, 7) have suggested that the time of introduction also plays a role in a variant acquiring sufficient fitness to invade a susceptible population. After the conclusion of wave 3, Delta, despite its known transmissibility (35) and immune evasion (36) characteristics, did not cause a fourth wave of infections in Colombia. This was likely due to its late arrival relative to the arrival of Mu and the large number of Mu infections likely having depleted the susceptible population for Delta. In contrast, when Mu was exported to countries where Delta was already established, Mu was unable to establish significant transmission chains.

Like other endemic human respiratory coronaviruses (i.e., strains NL63, OC43, 229E, and HKU1), SARS-CoV-2 will likely settle into seasonal cycles in which variants will continue to emerge and displace each other without causing severe mortality (37, 38). An understanding of this natural process of variant competition (emergence → displacement → establishment) is required to apply appropriate control measures as the pandemic inevitably transitions into an endemic situation. Others have recognized that the emergence of variants is linked to the accumulation of excess mutations in immunocompromised people (and thus violation of the molecular clock), as well as reverse zoonosis events (39–43). Here, we demonstrate that variant displacement events occur through an increase in transmissibility (even in short time windows) or immune evasion characteristics or a mixture of both. This finding supports the recommendations that COVID-19 vaccine boosters should be periodically reevaluated based on the most recently circulating variants, as is done for seasonal influenza (44). After being introduced at an opportune time when environmental factors favor its precise genetic profile, the successful establishment of a variant in a susceptible population requires an increase in fitness through an increase in genetic diversity and effective reproduction rate (45). This will be manifested by an increased number of cases and morbidity, and thus, continued diagnostic testing will be required, together with continued genomic surveillance to assess variant prevalence and mutational composition.

To study the entire process of SARS-CoV-2 variant emergence and competition, it is incumbent upon the molecular epidemiology community to adopt standardized methodologies (46). While Bayesian approaches are attractive, they demand more computational investment and expertise and may be limited by large data sets (47). Thus, continued investment in state-of-the art hardware and novel evolutionary models and inference methods will enable reliable guidance to be provided in a timely manner. At no point in history has research output and interconnectedness among infectious disease scientists been higher than during the COVID-19 pandemic. However, continued strengthening of communication and sharing of resources among key stakeholders will be vital to prepare for the next pandemic.

## MATERIALS AND METHODS

### Epidemiological and spatial data.

National-level counts of COVID-19 cases, deaths, estimates of *R_e_*, numbers of vaccinated individuals, and government measure stringency indices in Colombia were retrieved from publicly released data provided by Our World in Data (OWID), accessible through the repository (https://ourworldindata.org/coronavirus, accessed on 7 February 2022). OWID builds its database using data supplied by public entities like government health authorities. Additionally, department-level (adm1) data on population, cases, deaths, vaccine administrations, and vaccine sourcing were obtained from the Datos Abiertos portal on the Colombian government website (https://www.datos.gov.co/browse, accessed on 7 April 2022).

Shapefiles for constructing vector maps at the adm1 level were obtained from Natural Earth (https://www.naturalearthdata.com/downloads/10m-cultural-vectors/, accessed on 17 February 2022). Conversion of the plain-text collection location of each viral genome to latitude and longitude values was obtained through the Nominatim geocoding application programming interface (API) utilizing OpenStreetMap data (https://nominatim.org/, accessed on 8 October 2021).

### Specimen collection.

Human respiratory specimens (nasal swabs and nasopharyngeal washes) were obtained from routine COVID-19 surveillance through the public health laboratories network of the Department of Antioquia, led by the National Institute of Health (INS) and by the Public Health Laboratory of the Department of Antioquia (PHLA). As part of the COVID-19 surveillance network, the One Health Genomic Laboratory (OHGL) at the Universidad Nacional de Colombia received specimens to perform genomic surveillance and routine testing. Standard operating procedures were followed for specimen collection, transport, and storage (48). Sample metadata were collected and submitted along with the testing specimens. The metadata obtained included self-reported epidemiological, demographic, and clinical data, such as age, gender, origin, date of onset of symptoms, date of sample collection, symptoms, comorbidities, hospitalization record, history of vaccination, recent travel, and clinical outcome (e.g., hospitalized, deceased, or nonhospitalized). From July 2020 to October 2021, 888 specimens (mostly from Antioquia) were submitted to the OHGL for SARS-CoV-2 routine molecular testing and sequencing.

### Molecular testing.

Molecular testing was performed as described previously (49). Briefly, RNA extraction was performed on respiratory specimens using the ZR viral kit (Zymo Research, Irvine, CA, USA) following the manufacturer´s instructions. Real-time reverse transcription-PCR (RT-PCR) was performed in a CFX96 thermocycler (Bio-Rad Laboratories, Hercules, CA, USA) using the iTaq universal probes one-step kit (Bio-Rad, Hercules, CA, USA) and specific SARS-CoV-2 primers/probe targeting the envelope and RNA-dependent RNA polymerase genes (50). In addition, amplification of the housekeeping human RNase P gene was included as an endogenous control for each sample using primers and probe reported elsewhere (51). The thermocycling conditions were 50°C for 10 min, 95°C for 3 min, 40 cycles of 95°C for 15 s, and 58°C for 30 s. Positive and negative (nontemplate) controls were included in each testing run. Samples were considered positive for SARS-CoV-2 when the cycle threshold (*C_T_*) value was ≤38.

### Whole-genome sequencing.

Leftover SARS-CoV-2 PCR-positive respiratory specimens were used for virus sequencing. Samples resulting in a *C_T_* value of ≤27 were selected for whole-genome sequencing, done by modifying a previously reported protocol (52). Briefly, reverse transcription was performed using the LunaScript RT supermix kit (New England Biolabs, Ipswich, MA, USA). Multiplex PCR for library preparation was performed using the SARS-CoV-2 ARTIC Network V2/V3 amplicon set. Amplicon pools were quantified using the Qubit double-stranded DNA (dsDNA) high-sensitivity (HS) assay kit (Thermo Fisher Scientific, Waltham, MA, USA). Prepared DNA was loaded onto an R9 flow cell (Oxford Nanopore Technologies, Oxford, UK) and run in a MinION sequencer (Oxford Nanopore Technologies, Oxford, UK). Bioinformatics analysis of raw sequencing data was performed following a protocol described elsewhere (53). Additionally, sequences were analyzed using the bioinformatics tools Pangolin (54), Nextclade (55), and Nextstrain (56). Seven specimens did not yield full genomes, and therefore, a total of 881 full genomes were characterized.

### Public genomic data set collection, filtering, and subsampling.

A schematic of the public data collection scheme is shown in Fig. S11. Following the criteria and methodologies from our previous study (45), we conducted the selection, filtering, and subsampling of SARS-CoV-2 sequences to be used in the current study. Briefly, within the time frame of the study (December 2019 to August 2021), we retrieved a globally representative set of 2,514 SARS-CoV-2 genomes (with the Latin American-Caribbean region [LAC] excluded) based on the daily Nextstrain v.8 (https://nextstrain.org) build on 15 September 2021. Nextstrain (56) uses an algorithm to down-sample the entire GISAID database in a representative manner down to ~3,000 sequences for accelerated analyses: in v.8, the down-sampling was performed such that there was a 2:1 ratio of recent/early sequences, where “recent” was defined as being collected less than 4 months prior to the access date (Fig. S4). In this study, the LAC region is taken to mean all countries in North America, South America, and the Caribbean except the United States and Canada.

In addition, all 69,124 SARS-CoV-2 sequences and their metadata with an origin in any LAC country except Colombia were pulled from the same local GISAID repository, also on 15 September 2021. The LAC sequences were arranged in increasing order of collection date and then randomly down-sampled by 65%, maintaining the collection date distribution, resulting in 24,498 sequences (Fig. S4). The 881 sequences collected in this study were added to 2,340 sequenced at other institutions in Colombia (3,221 total) and to the global sequences described above, resulting in a combined initial data set of 30,233 sequences (henceforth called data set G). Thus, data set G contains a global distribution of sequences with an overrepresentation of sequences from LAC and all sequences from Colombia.

An additional data set focused on VOI Mu (data set Mu) was constructed. All genomes, regardless of country, classified as Mu or Pango lineages B.1.621 and B.1.621.1 and collected before October 2021 were pulled from a local copy of the GISAID repository on 14 March 2022. This initial data set, which we call data set Mu, contained 12,388 sequences.

Each data set was analyzed using a local, sandboxed Docker deployment of Nextclade (55, 57). Genomes with “bad” quality control (QC) scores (using the default scoring metrics in Nextclade) or >50 total mutations were discarded. As described previously (45), the filtered sequences within each data set were then aligned, clipped, and deduplicated such that the most ancestral sequence of an identical set was retained. Quality control and deduplication resulted in a data set G containing 17,861 sequences, of which 2,906 were of Colombian origin, 13,829 were from LAC but of non-Colombian origin, and 1,126 were of non-LAC origin. Quality control and deduplication also resulted in data set Mu containing 5,060 unique genomes derived from all over the world.

### Phylogenetic and molecular clock evaluation.

Data sets G and Mu were iteratively refined through rounds of maximum-likelihood (ML) phylogenetic tree inference, followed by temporal signal outlier removal, as described previously (45). The process was repeated until no more outliers were detected using either TempEst (58) or TreeTime (59). This process further reduced data set G to 17,036 sequences and data set Mu to 5,023 sequences.

To create initial trees for the Bayesian phylodynamic analyses, time-scaled phylogenies were estimated from the ML tree topologies and collection dates using TreeTime. All ML and time trees obtained were visualized using the ggtree (60) package in R, and the root-to-tip regressions were visualized using the ggplot2 v.3.4 (61) package in R (v.4.1) (Fig. S12 and S13).

### Temporal dynamics of the main lineages of SARS-CoV-2 in Colombia.

Data set G was used for a time-scaled phylogenetic reconstruction using Bayesian inference through Markov chain Monte Carlo (MCMC) in BEAST (62). The time-scaled phylogenies obtained as described above were used as the starting tree for an alternative likelihood function, together with a constrained model implemented in BEAST v.1.10.5pre_thorney_v0.1.1 (https://github.com/beast-dev/beast-mcmc/releases/tag/v1.10.5pre_thorney_v0.1.1) and a nonparametric Skygrid coalescent prior (63). MCMC analysis was performed for 4 × 10^8^ iterations, sampled every 4 × 10^5^ steps for both log and tree files. Calculations were considered complete when the effective sample size (ESS) values for all parameters were ≥250 (a measurement of convergence) as assessed by Tracer v.1.7 (64).

A maximum-clade-credibility (MCC) tree was generated using TreeAnnotator v.1.10.4 after removing a 10% burn-in as determined by Tracer. The reference sequence Wuhan-Hu-1 (GenBank accession number NC_045512 or GISAID accession number EPI_ISL_406798) was used as a root for the obtained MCC tree. The rooted MCC tree was visualized using ggtree v.3.0 (60) in R.

### Genetic demographic analysis and estimation of the rates of infections.

We next sought to determine the levels of genetic diversity and effective reproduction rates among the SARS-CoV-2 lineages circulating in Colombia with evidence of local diversification and diffusion. For this purpose, major monophyletic clades primarily composed of Colombian sequences were identified through inspection of the MCC tree phylogenetic analysis of data set G (Fig. 1D). Briefly, collection country metadata were mapped to the MCC tree and monophyletic clades composed of >90% Colombian taxa were identified (Fig. S7) following the method of Dellicour et al. and Hong et al. (65, 66). The sequences corresponding to those clades were extracted into separate nucleotide alignments. Non-Colombian sequences were removed, and any alignments containing fewer than 10 total Colombian sequences were ignored. These selection criteria resulted in 12 lineages being selected for further analysis.

Bayesian skyline plots (BSPs) were estimated for Colombian sequences from those clades denoted in Fig. S7 to infer the population dynamics of SARS-CoV-2 in terms of changing levels of relative genetic diversity (*N_e_τ*) through time (67). MCMC analysis was performed with BEAST2 v.2.6.7 (68) for 1 × 10^9^ chains sampled every 1 × 10^5^ steps for both log and tree files. In all cases, an uncorrelated exponential distribution (UCED) relaxed molecular clock was used, with a dimension of 10 for the group size. Calculations were considered complete when the ESS values for all parameters were ≥250 (a measurement of convergence) as assessed by Tracer v.1.7 (64). The BSPs were visualized using ggplot2 (61) in R.

We utilized a birth-death susceptible-infectious-recovery (BDSIR) model (69), implemented in BEAST2 v.2.6.7 (68), to estimate the effective reproduction rates (*R_e_*s) of the same sequences selected for BSPs, similar to our previous work (45). This model allows the estimation of *R_e_*, the rate of an infection being transmitted (λ) or becoming noninfectious (δ), and the probability that an infectious individual was sampled during the study (*s*). Briefly, the following priors were used: a population size of susceptible individuals was fixed to a gamma distribution with α = 10^2^ and β = 4 × 10^4^, δ was set considering the duration of the infectious period of a SARS-CoV-2 infection (70) (exponential, mean = 40), and the standard deviation was set at 1.3. The strict molecular clock model and substitution model HKY+G were selected, and MCMC chains were run for 8 × 10^7^ generations sampled every 8 × 10^4^ steps. Calculations were considered complete when the ESS values for all parameters were ≥250 (a measurement of convergence) as assessed by Tracer v.1.7 (64). Epidemiological information from the BDSIR model was extracted by applying the plot_BDSIR.R script described by the tutorial available at https://www.beast2.org/tutorials/.

### Continuous phylogeographic analysis.

To determine the diffusion of lineages across Colombia, we applied a continuous phylogeographic inference, utilizing a flexible relaxed random walk (RRW) model with a Cauchy distribution to determine the among-branch heterogeneity in diffusion velocity (13) with an estimated time-scaled tree as the initial input. We first divided the monophyletic Colombian clades, shown in Fig. S6, by epidemic wave. Only monophyletic Colombian clades appearing in waves 2 and 3 were included in the analysis because there was evidence of local diversification and diffusion (as opposed to significant external importation, such as that observed in wave 1; see “Data availability” below for BEAST logs), as described in Dellicour et al. (65): briefly, the introduction of each lineage into Colombia is represented by its oldest Colombian sequence. Since the analysis only includes Colombian monophyletic clades, it does not consider external introduction events. Each sequence was associated with the latitude and longitude of its collection location; due to uncertainty and to avoid duplicated sampling locations, we added a jitter window with a size of 0.01, as suggested by Dellicour et al. (14). MCMC chains were run in BEAST v.1.10.4 (62) for 10^8^ generations and sampled every 10^6^ steps. Calculations were considered complete when the ESS values for all parameters were ≥250 (a measurement of convergence) as assessed by Tracer v.1.7 (64). The R package SERAPHIM v1.0 (15) was used to extract and statically map spatiotemporal information embedded in the posterior trees.

### Estimating transmissibility and immune evasion advantages.

Using the metadata extracted from 2,038 SARS-CoV-2 genomes collected in Colombia with collection dates between 1 January 2021 and 1 July 2021 (the transition from wave 2 to wave 3), we utilized a set of previously described models (17, 18) to understand the relationship between increases in transmissibility and immune evasion characteristics of the variants present during the third epidemic wave. Briefly, a multinomial logistic regression model was first used to estimate the maximal growth advantages of (i) the main wave 3 lineages (Alpha, Gamma, Lambda, and Mu) treated as a single group over the main wave 2 lineages (B.1, B.1.111, B.1.420, and B.1.1.348) treated as a single group and (ii) Alpha, Gamma, and Mu individually against each other and the nonvariant lineages (competition between Lambda and the other variants was not considered due to the low sequence count). We then applied a second model that defines the growth advantage between any two variants as being dependent on increases in transmissibility, the level of immune evasion, and the level of seroprevalence in the population. The model was algebraically solved for the immune evasion level using the growth advantages calculated from the first model as the known-input parameter and transmissibility increase and population seroprevalence as independent variables. Confidence intervals (95%) were computed to propagate uncertainty. We assume the generation time of the virus to be normally distributed with a mean of 5.2 days and a standard deviation of 0.8 days (71).

### Discrete phylogeographic analysis of Mu.

To analyze the origin and dispersal of Mu, a discrete-trait phylogeographic inference was performed on data set Mu using an incorporated travel history model described previously (25, 72). Since the magnitude of the data set (>2,000 sequences) would require significant calculation time to achieve a fully Bayesian approach using an incorporated travel history model, we followed a subsampling strategy to shrink the data set (66). Briefly, TreeTime (59) was used to obtain an initial time-stamped phylogeny, followed by an ML-based discrete ancestral character inference to characterize the geographic structure of the topology obtained. Since monophyletic clusters consisting solely of sequences from the same country do not supply any additional information regarding the between-state spreading process, we eliminated all but one randomly selected sequence per monophyletic cluster (66). The resulting refined data set Mu consisted of a total of 1,828 sequences, which was more practical for a fully Bayesian phylogeographic inference (Fig. S13). A Python script was used to incorporate any travel history included in the associated metadata, and the Bayesian discrete-state phylogeographic reconstruction was performed as described previously (72). MCMC analysis was run for 2 × 10^8^ chains, sampled every 2 × 10^4^ chains using BEAST v.1.10.5 (62). Calculations were considered complete when the ESS values for all parameters were ≥250 (a measurement of convergence) as assessed by Tracer v.1.7 (64). A *post hoc* analysis to determine the Markov jump estimates of transition histories was generated using the BEAST tree sampling tools TaxaMarkovJumpHistoryAnalyzer and TreeMarkovJumpHistoryAnalyzer, available from the BEAST codebase (https://github.com/beast-dev/beast-mcmc). The R packages MarkovJumpR (72) and circlize (73) were used for visualization purposes.

This work conformed to the Declaration of Helsinki. Molecular testing and sequencing were conducted after written informed consent obtained from patients at the time of the medical consult at each of the collaborating health care facilities in the public health network of the Antioquia Department. This work was conducted as part of the Colombian Public Health Surveillance System and followed the World and Pan-American Health Organization guidelines on ethics in public health surveillance (https://iris.paho.org/bitstream/handle/10665.2/34499/9789275319840-spa.pdf?sequence=6, accessed on 4 January 2021) and was approved by the ethics committee of the Corporación de Investigaciones Biológicas (CIB; no. 26072021) in Medellín.

### Data availability.

All R scripts and underlying data used to generate figures will be provided upon request after publication of the manuscript. BEAST XML files and logs are available at the following repository: https://doi.org/10.5281/zenodo.7806522. The 888 SARS-CoV-2 sequences generated in this study have been deposited to GenBank with the following accession numbers: OQ551203 to OQ551313, OQ557515 to OQ557625, OQ557827 to OQ557937, OQ558912 to OQ559022, OQ564650 to OQ564865, OQ564867 to OQ564972, OQ565007 to OQ565116, and OQ572724 to OQ572735.
