# Supplementary material for: The Principles of SARS-CoV-2 Intervariant Competition Are Exemplified in the Pre-Omicron Era of the Colombian Epidemic
Source: Microbiol Spectr. 2023 May 16;11(3):e05346-22. doi: 10.1128/spectrum.05346-22 (PMC10269686; doi:10.1128/spectrum.05346-22)
Supplement: Supplemental file 1 — Supplemental material. Download spectrum.05346-22-s0001.pdf, PDF file, 5.4 MB [file spectrum.05346-22-s0001.pdf]

Supplementary Information for:

The principles of SARS-CoV-2 inter-variant competition are exemplified in the pre-Omicron era of the Colombian epidemic

Gregory S. Orf[1,2,‡,*], Lester J. Pérez[1,2,‡], Karl Ciuoderis[2,3], Andrés Cardona[2,3], Simón Villegas[2,3], Juan P. Hernández-Ortiz[2,3], Guy Baele[4], Aurash Mohaimani[1,2], Jorge E. Osorio[2,3,5], Michael G. Berg[1,2], Gavin A. Cloherty[1,2]

[1] Infectious Disease Research, Abbott Diagnostics Division, Abbott Laboratories, Abbott Park, IL 60064, USA

[2] Abbott Pandemic Defense Coalition (APDC)

[3] UW-GHI One Health Colombia, Universidad Nacional de Colombia Sede en Medellín, Medellín, Colombia

[4] Laboratory of Clinical and Evolutionary Virology, Department of Microbiology, Immunology and Transplantation, Rega Institute, KU Leuven, Leuven, Belgium

[5] UW-GHI One Health Colombia, University of Wisconsin-Madison, Madison, WI 53706, USA

[‡] equal contribution

[*] to whom correspondence should be addressed: gregory.orf@abbott.com

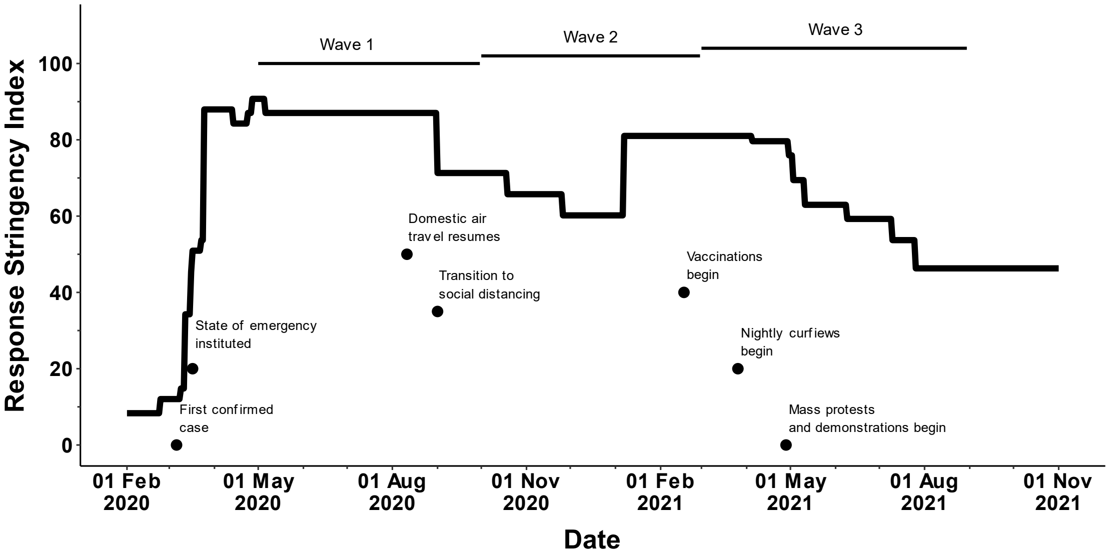

Fig. S1: Response stringency index during the COVID-19 epidemic in Colombia. The stringency index is a measure of the strictness of government actions for mitigating COVID-19 (available from Our World in Data, https://ourworldindata.org/covid-stringency-index). Selected public events and pandemic mitigation efforts are shown on the timeline.

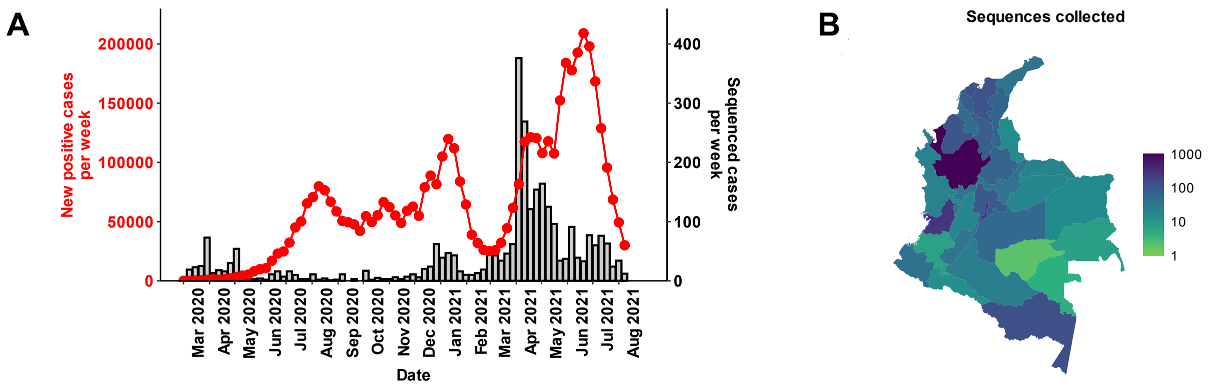

Fig. S2: Sub-national genome sequencing efforts during the first three local epidemic waves of SARS-CoV-2 in Colombia. A. The relationship between weekly positive tests and the number of those positive cases that resulted in the collection of a full viral genome sequence. **B.** The results of sequencing efforts at the Department (*adm1*) level during the timeframe of the study.

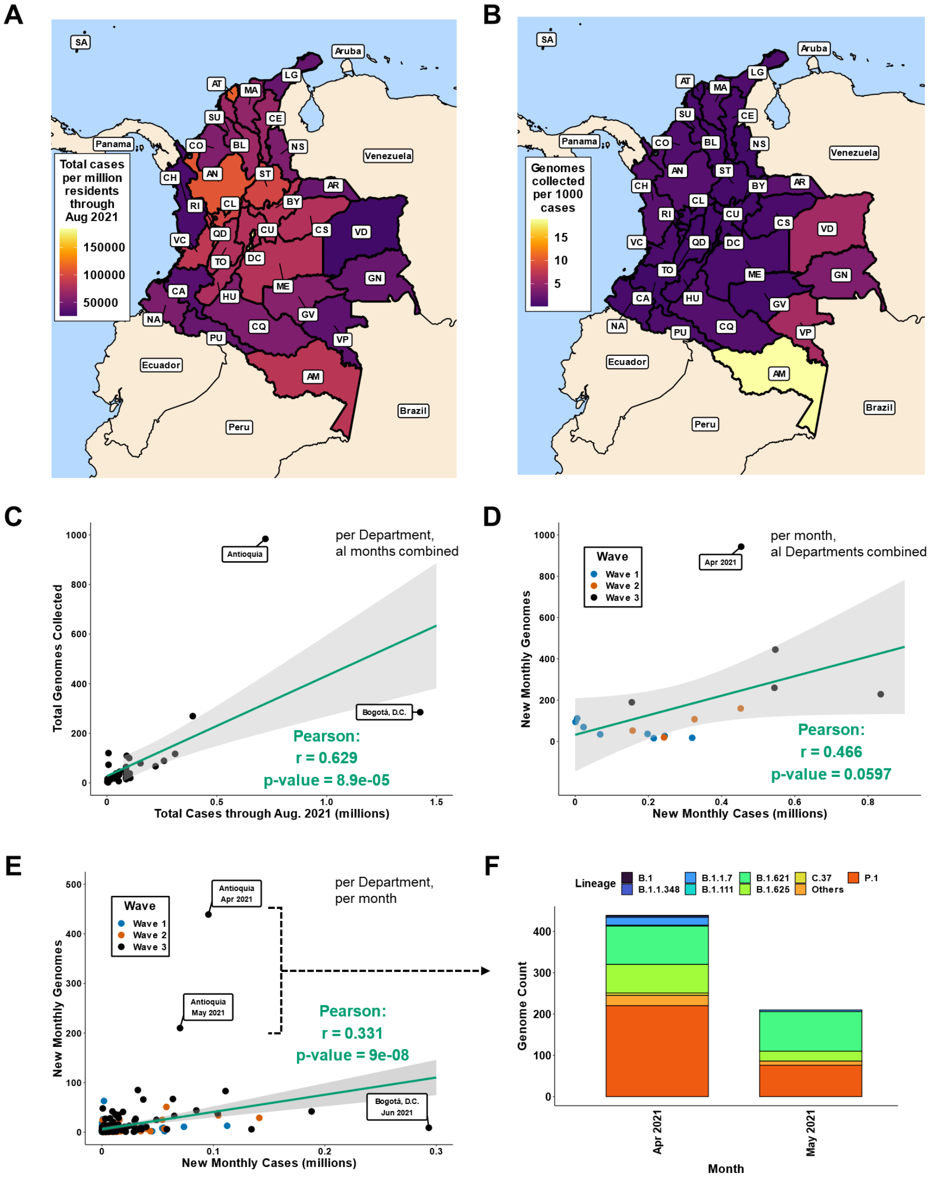

Fig S3: Spatiotemporal statistics indicating positive correlation between SARS-CoV-2 infection count and genome collection count in the 33 Departments of Colombia from March 2020-August 2021. A. Cumulative SARS-CoV-2 cases per million residents of each Department. **B.** Cumulative genomes collected per 1,000 reported cases of SARS-CoV-2 infection. **C.** Cumulative genomes collected in each Department over the study timeframe plotted against the total case count with linear regression and Pearson bivariate correlation test. **D.** Monthly genome collections plotted against the monthly SARS-CoV-2 case count (all Departments combined) with linear regression and Pearson bivariate correlation test. **E.** The same analysis as panel D, except that each Department is broken out separately. **F.** In-depth analysis of the sequencing efforts in the Department of Antioquia during April-May 2021, which are spatiotemporal outliers observed in panel E. Though Antioquia is oversampled in these two months, the shift from a Gamma-dominated epidemic to a Mu-dominated epidemic is evident, and matches the shifts seen in other Departments (see Fig. S4).

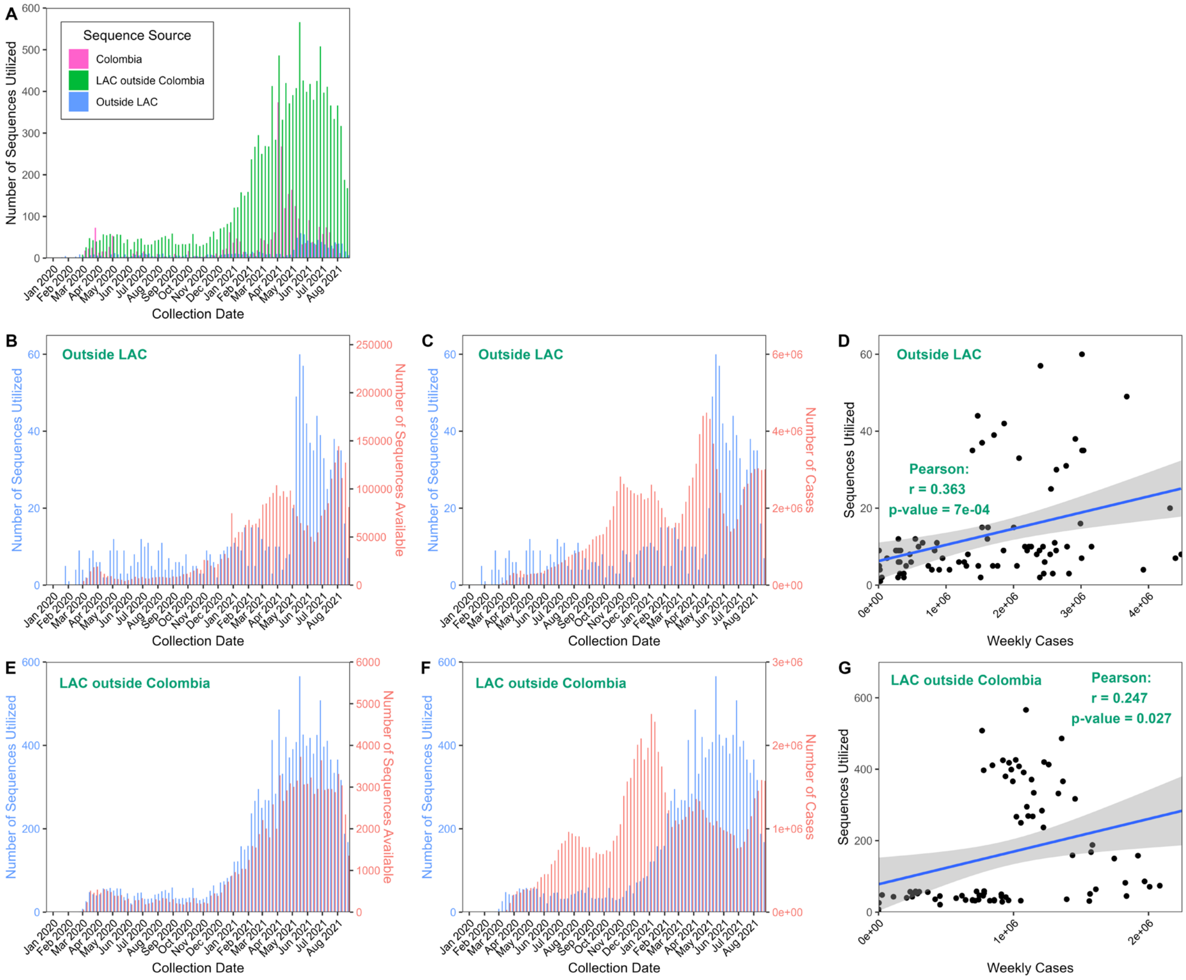

Fig S4: Spatiotemporal statistics indicating positive correlation between SARS-CoV-2 infection count and genome utilization in the study period between March 2020-August 2021. A. Genomes utilized for the study binned by collection week and stratified by geographic area. **B.** Number of sequences utilized and number of sequences available from countries outside the LAC region, binned by week. **C.** Number of sequences utilized and number of cases from countries outside the LAC region, binned by week. **D.** Weekly case count outside the LAC region plotted against the utilized LAC region SARS-CoV-2 genomes collected during that week, with linear regression and Pearson bivariate correlation test. **E-G.** The same analyses as panels **B-D** performed on sequences sourced from the LAC region outside of Colombia.

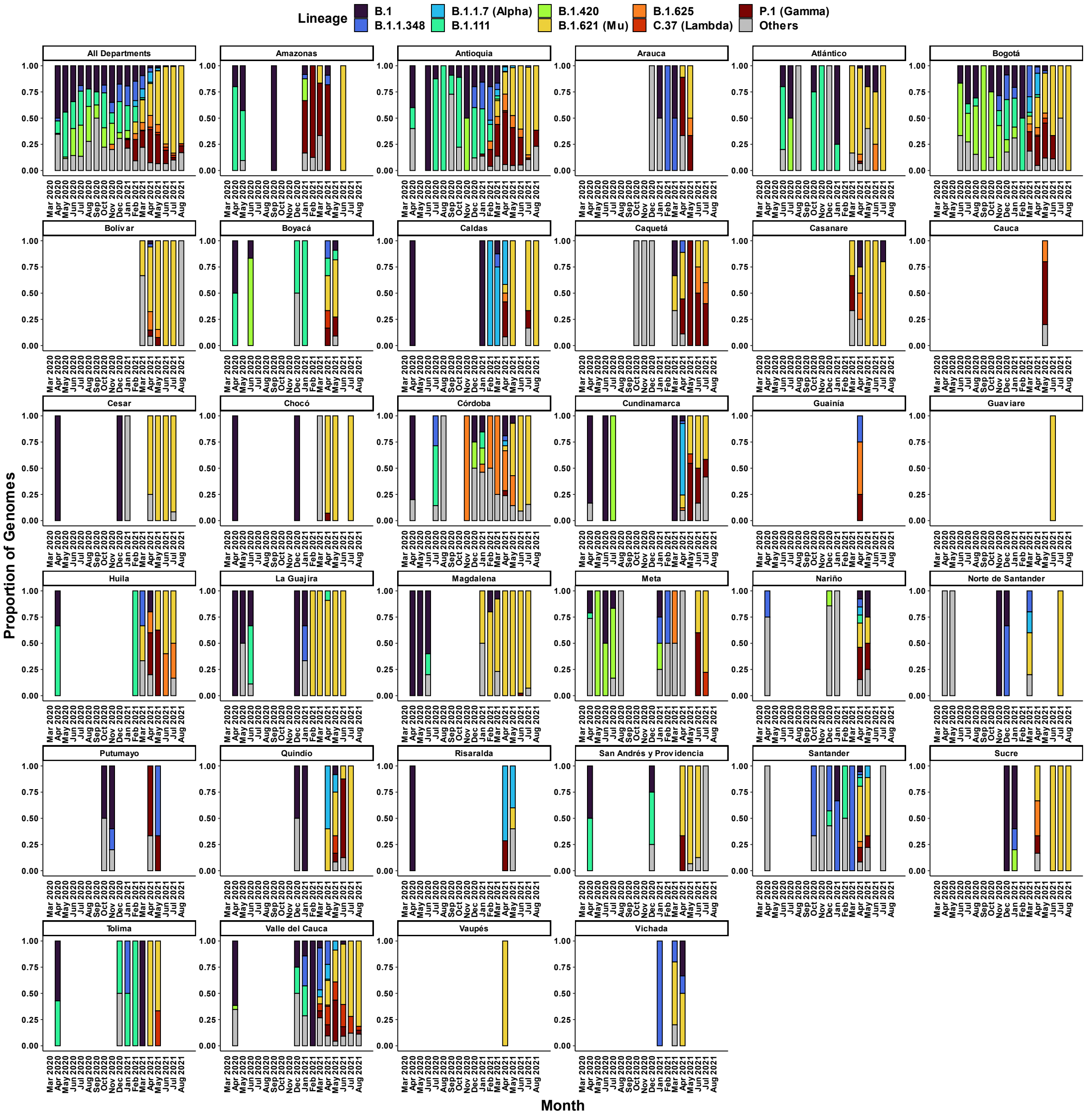

Fig S5: Time-resolved PANGOLIN lineage distribution at the sub-national level. 2,864 total sequenced genomes collected across the thirty-three Departments of Colombia are represented.

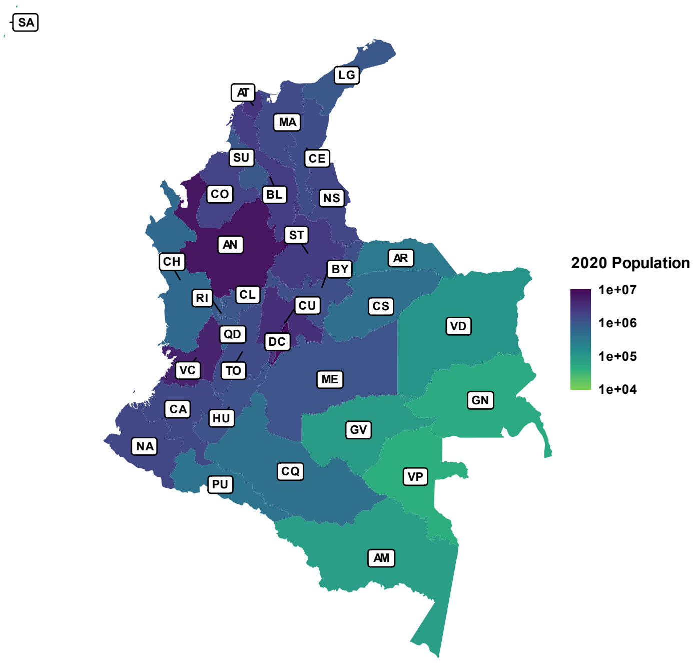

Fig. S6: Population distribution in Colombia at the *adm1* (Department) level. Population is colored on a logarithmic scale.

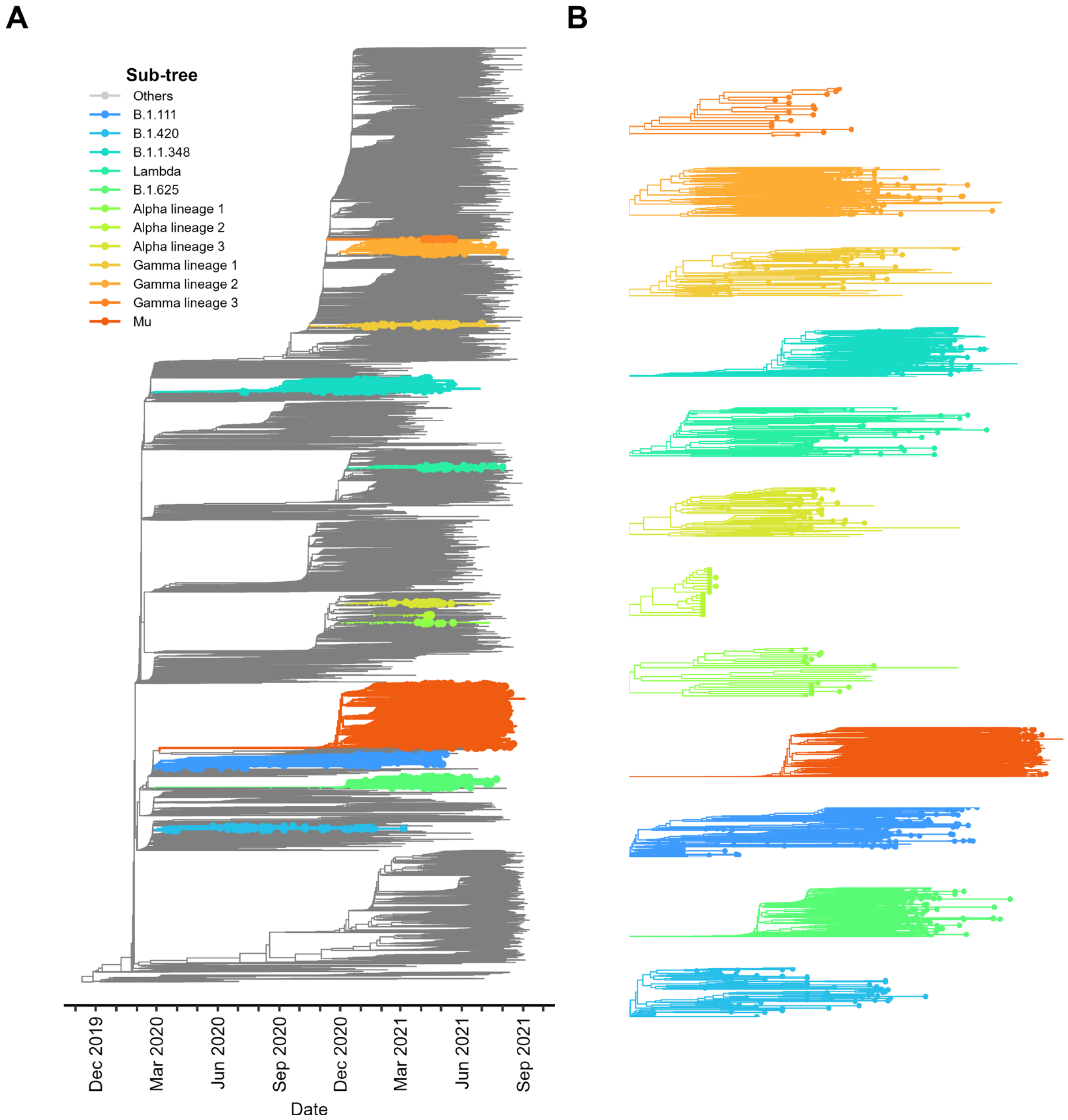

Fig. S7: Colombia-majority monophyletic clades identified from the MCC tree presented in Fig. 1 of the main text. (A) The entire MCC tree is shown with branches colored by identified group. (B) The identified clades are magnified for detail. Tips denoted by dots are from Colombia; the sequences corresponding to these tips were extracted for the demographic reconstruction presented in Fig. 3 of the main text.

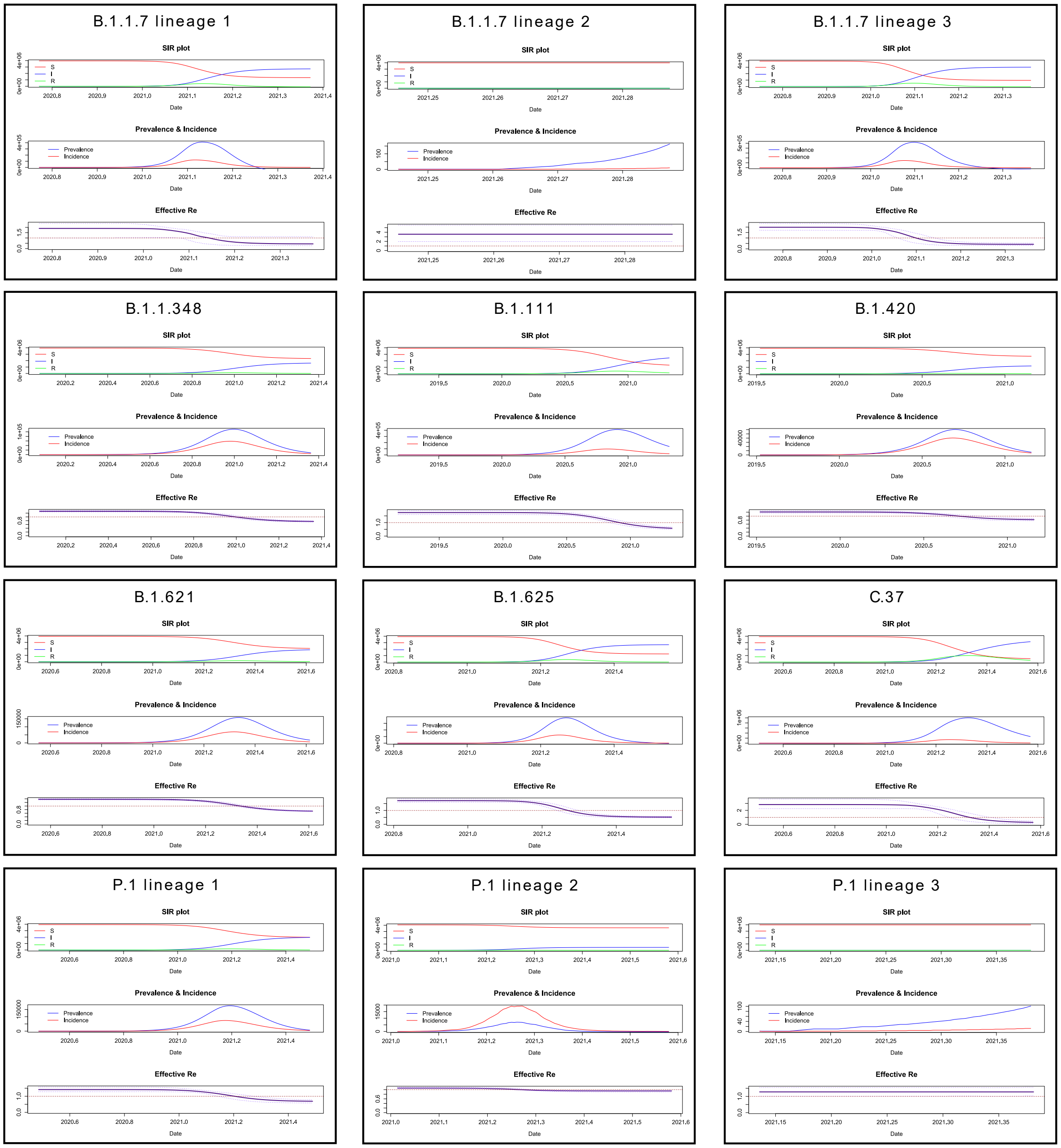

Fig. S8: Calculated incidence/prevalence and SIR trajectories of major SARS-CoV-2 lineages during the first three waves of the local epidemic in Colombia. The incidence/prevalence and SIR trajectories reveal at which stage in the epidemic each cluster was sampled.

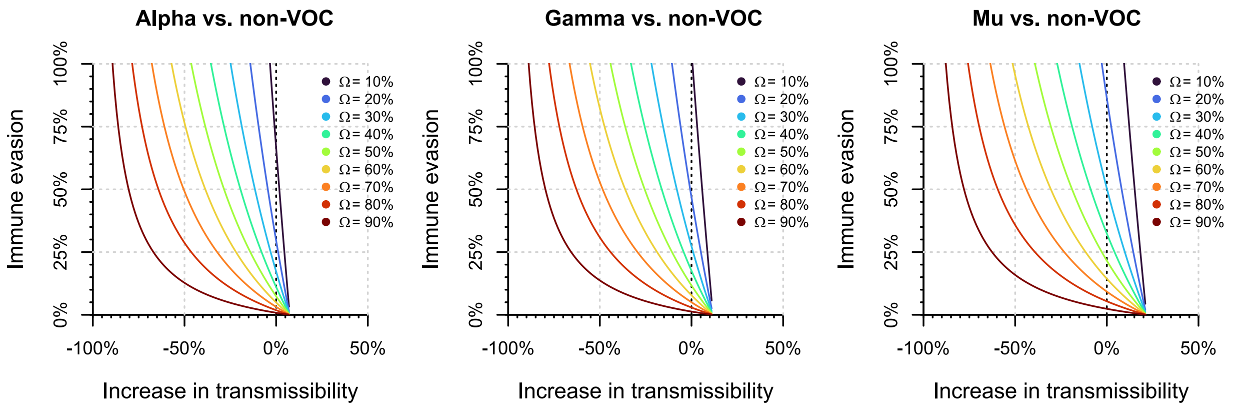

Fig. S9: Epidemiological modeling of the competition of Alpha, Gamma, and Mu against non-VOC lineages during wave 3 of the local SARS-CoV-2 epidemic in Colombia. The level of population seroprevalence (*Ω*) against non-VOC lineages influences the relationship between increases in transmissibility and immune evasion character for the variants entering the population against the backdrop of the pre-existing non-VOC lineages. The models are generated based on the daily growth advantage of each variant over non-VOC lineages between March-June 2021 that is calculated from the observed changes in variant proportions over time.

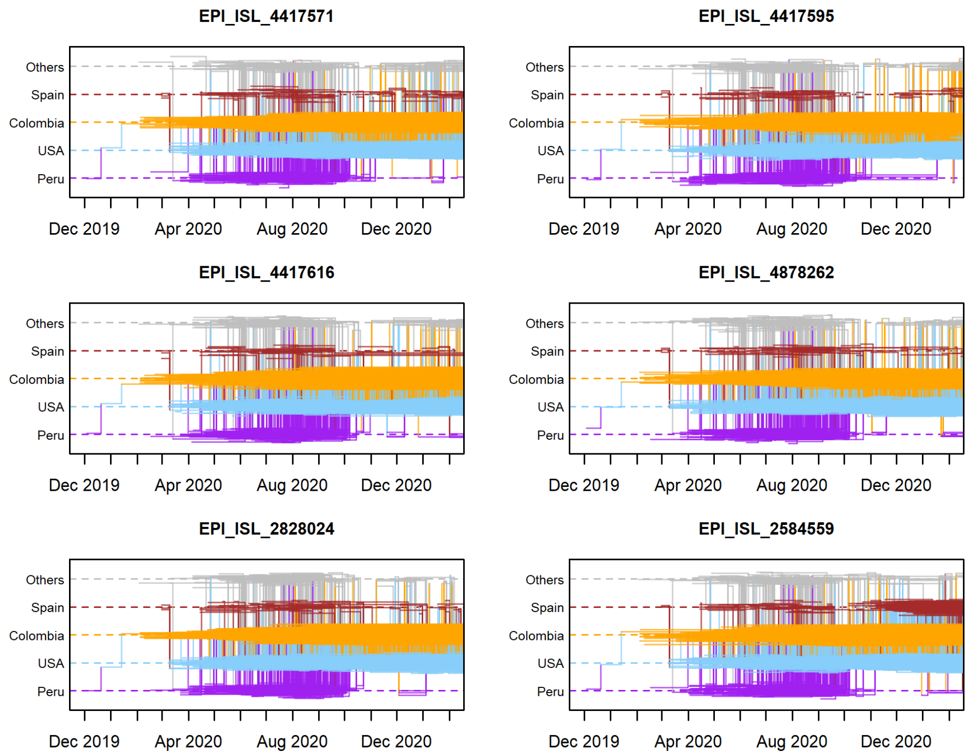

Fig. S10: Markov jump trajectory plots of the ancestral transition history for the most ancestral genome from Colombia for each major clade of Mu. The trajectories are summarized from a posterior tree distribution with Markov jump history annotation using a sampling location and travel history model. Lines in a horizontal trajectory represents the time during which a particular location state is maintained in the spatiotemporal ancestry of the virus, and vertical lines represent Markov jumps between two locations in the trajectory.

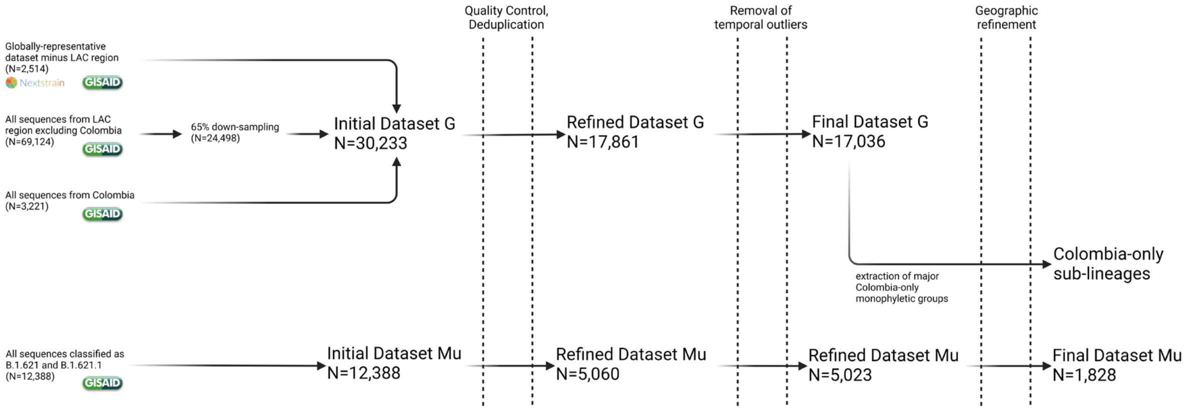

Fig. S11: A schematic illustrating the genomic collection, subsetting, and filtering steps undertaken in this study to produce the analyzed datasets. The date of the pulls from the GISAID database are denoted in the main text.

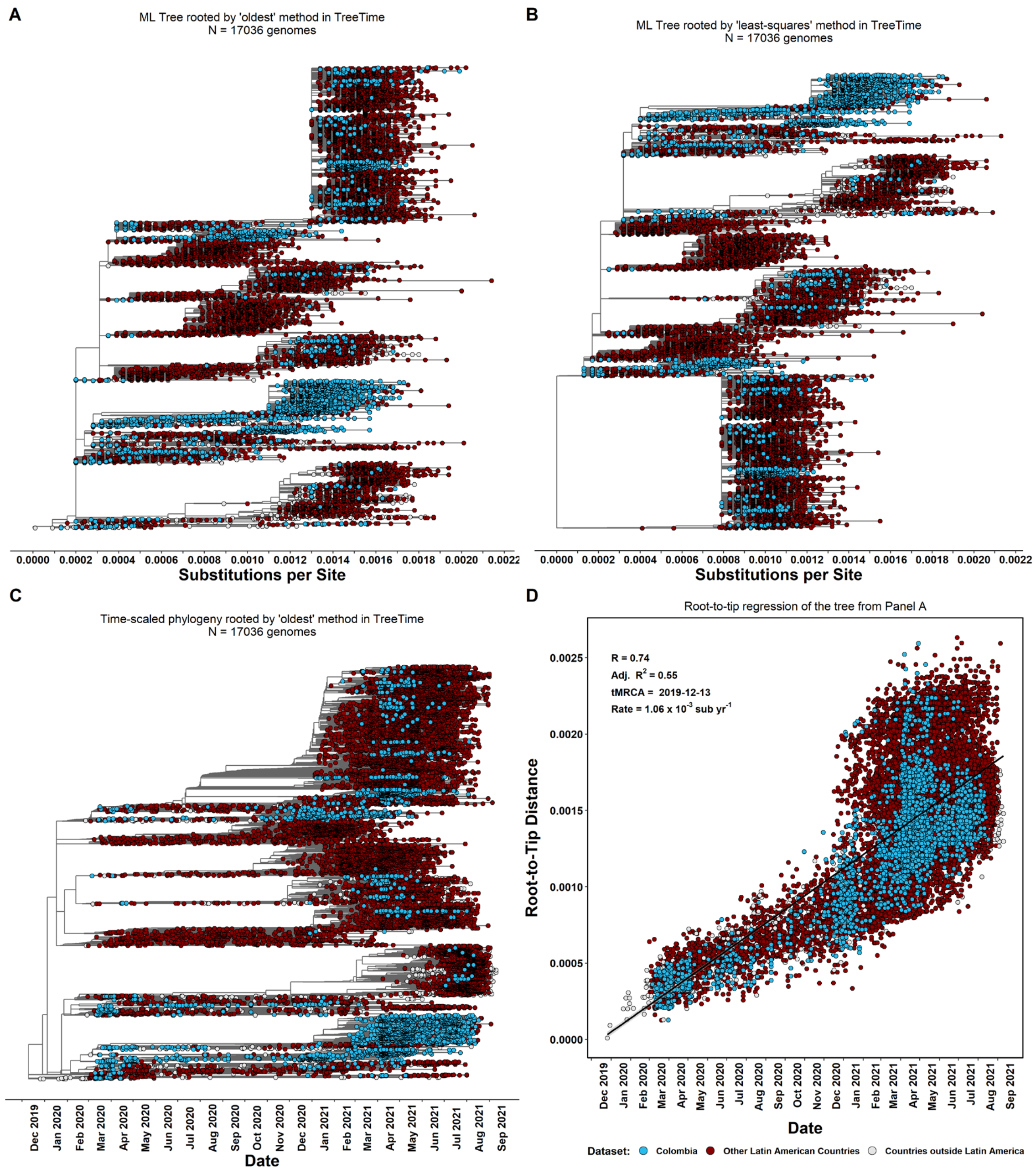

Fig. S12: ML trees, time-scaled phylogenies, and root-to-tip regression of genetic distances and sampling dates for Dataset G. A. and B. An ML tree estimated from N = 17,036 sequences rooted based on the oldest sequence or least-squares method, respectively. **C.** A time-tree (ML tree scaled using collection dates) using the ML tree from panel A as the starting tree. **D.** A root-to-tip regression of genetic distance and sampling dates based on the ML tree obtained in panel A, showing a positive molecular clock signal. In all panels, the tips are color-coded based on sampling region.

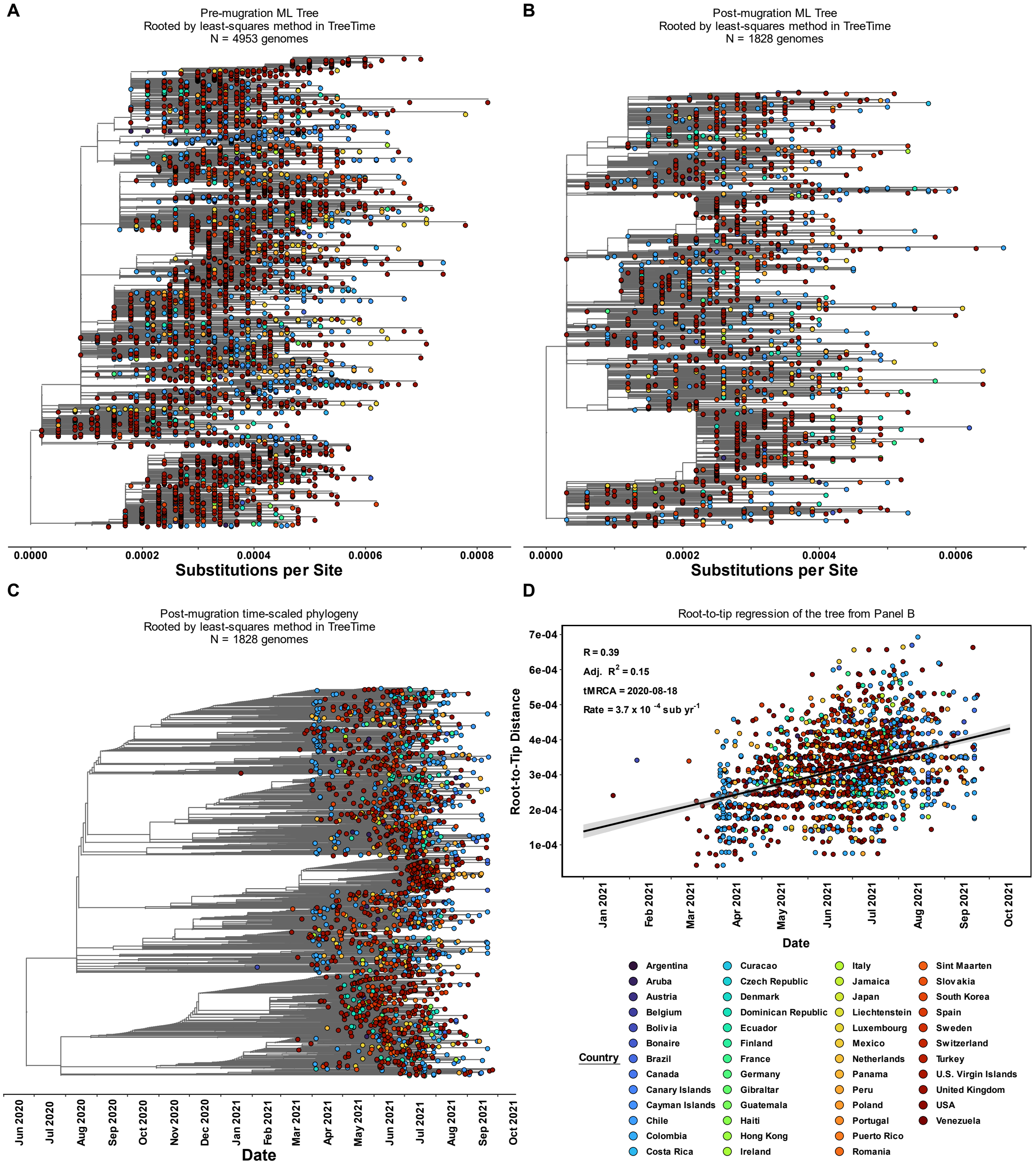

Fig. S13: ML trees, time-scaled phylogenies, and root-to-tip regression of genetic distances and sampling dates for Dataset Mu. A. An ML tree estimated from N = 4,953 sequences rooted based on the least-squares method, respectively. **B.** An ML tree estimated from a subset of N = 1,828 sequences from Panel A. The subsetting was achieved through the process of collapsing monophyletic groups composed of the same sampling country down to a single representative. A time-tree (ML tree scaled using collection dates) using the ML tree from panel B as the starting tree. **D.** A root-to-tip regression of genetic distance and sampling dates based on the ML tree obtained in panel B, showing a positive molecular clock signal. In all panels, the tips are color-coded based on sampling country.

Table S1: Spike subunit S1 mutations found in variants present in Colombia during the study period. The list is not exhaustive and only describes the minimum lineage-defining mutations in each variant. Spike region abbreviations: NTD: N-terminal domain; RBD: receptor-binding domain; RBM: receptor-binding motif; SD1: sub-domain 1; SD2: sub-domain 2; S1/S2: furin cleavage site at S1/S2 boundary

**Spike Mutation**	**Region**	**Variant**	**Effect**
L18F	NTD	Gamma	Immune evasion (*1*)
T19R	NTD	Delta	Immune evasion (*2*)
T20N	NTD	Gamma	Immune evasion (*1*)
Δ69-70	NTD	Alpha	Transmissibility (*1*)
G75V	NTD	Lambda	Transmissibility (*3*)
T76I	NTD	Lambda	Transmissibility (*3*)
D138Y	NTD	Gamma	Unknown
Δ144	NTD	Alpha	Immune evasion (*1*)
Y144S	NTD	Mu	Immune evasion (*1*)
Y145N	NTD	Mu	Immune evasion (*1*)
Δ157-158	NTD	Delta	Immune evasion (*1*)
R190S	NTD	Gamma	Unknown
R246N	NTD	Lambda	Immune evasion (*1*)
Δ247-253	NTD	Alpha, Lambda	Immune evasion (*1*)
R346K	RBD	Mu	Immune evasion (*4*)
K417T	RBD	Gamma	Immune evasion (*5*)
L452Q	RBM	Lambda	Immune evasion (*6*)
L452R	RBM	Delta	Immune evasion and transmissibility (*6*)
L478K	RBM	Delta	Transmissibility (*5*)
E484K	RBM	Gamma, Mu	Immune evasion (*5*)
F490S	RBM	Lambda	Immune evasion (*1*)
N501Y	RBM	Alpha, Mu	Transmissibility (*5*)
A570D	SD1	Alpha	Transmissibility (*7*)
D614G	SD2	All	Transmissibility (*5*)
H655Y	SD2	Gamma	Transmissibility (*8*)
P681H	S1/S2	Alpha, Mu	Transmissibility (*5*)
P681R	S1/S2	Delta	Transmissibility (*5*)

Table S2. Dates of introduction or emergence of the twelve major SARS-CoV-2 lineages of interest. The clades, shown in Main Text, Fig. 3A, were extracted from the MCC tree shown in the Main Text, Fig. 1D. The median emergence date is the time of the most recent common ancestor (tMRCA) for each lineage, calculated using the median height of its ancestral node. 95% HPD Interval: highest posterior density interval with a fixed 95% probability.

Lineage	Median Emergence Date	95% HPD Interval
B.1.111	2020-02-26	2020-02-22 – 2020-02-27
B.1.420	2020-03-01	2020-02-25 – 2020-03-07
B.1.1.348	2020-03-01	2020-02-21 – 2020-03-15
Lambda	2020-12-19	2020-12-10 – 2021-01-05
B.1.625	2020-08-19	2020-06-24 – 2020-09-24
Alpha lineage 1	2020-12-13	2020-11-29 – 2020-12-21
Alpha lineage 2	2021-03-06	2021-02-16 – 2021-03-30
Alpha lineage 3	2020-12-19	2020-12-08 – 2021-01-13
Gamma lineage 1	2020-11-12	2020-10-24 – 2020-12-09
Gamma lineage 2	2020-12-12	2020-11-28 – 2020-12-24
Gamma lineage 3	2021-01-12	2020-12-05 – 2021-01-25
Mu	2020-06-24	2020-05-17 – 2020-08-12

Table S3. Dates at which the mean R_e value dropped below 1 for the twelve main monophyletic lineages of SARS-CoV-2 in Colombia calculated using the BDSIR model. The plots depicting the change in median R_e over time corresponding to these lineages are shown in the Main Text, Fig. 3C. *NA*: not applicable; the corresponding value did not drop below 1 during the timeframe utilized in Main Text, Fig. 3C. 95% HPD Interval: highest posterior density interval with a fixed 95% probability.

Lineage	Date at which R_e falls below 1	95% HPD Interval
B.1.111	2020-11-29	2020-11-05 – 2020-12-30
B.1.420	2021-02-14	2021-01-09 – 2021-04-03
B.1.1.348	2021-02-09	2021-01-21 – 2021-02-27
Lambda	2021-04-29	2021-04-02 – 2021-05-30
B.1.625	2021-04-13	2021-04-02 – 2021-04-23
Alpha lineage 1	2021-04-17	2020-11-30 – *NA*
Alpha lineage 2	*NA*	*NA*
Alpha lineage 3	2021-04-26	2021-04-10 – 2021-05-09
Gamma lineage 1	2021-04-09	2021-03-18 – 2021-05-04
Gamma lineage 2	2021-04-14	2021-04-14 – 2021-04-24
Gamma lineage 3	*NA*	*NA*
Mu	2021-05-21	2021-05-09 – 2021-06-01

Table S4. Impact of different environmental variables on the dispersal location of SARS-CoV-2 lineages from the locally propagating epidemic waves (wave 2 and wave 3) in Colombia. Bayes factors (BF) supporting the association between environmental variables and tree node locations are shown. Results were collected using the R-based package SERAPHIM (*9*) based on 100 posterior trees obtained by continuous phylogeographic inference. Each environmental raster was tested as a factor attracting ("A") or repulsing ("R") viral lineages. Following previous guidance(*10*), we consider a BF value >20 as strong support for a significant correlation between the environmental distances and dispersal durations (in bold). Vaccination was not considered a variable during wave 2 since vaccines were not available.

Environmental factor	All branches, Wave 2	All branches, Wave 3
Elevation (R)	0.0638	0
Elevation (A)	0.0526	**99**
Population density (R)	0.0101	0
Population density (A)	0	0
Daily temperature (R)	**49**	0.1236
Daily temperature (A)	0	4
Vaccination (R)	-	1.5641
Vaccination (A)	-	0.8182

Table S5. Impact of several environmental variables on the dispersal velocity of SARS-CoV2 lineages from the locally dispersed epidemic waves (wave 2 and wave 3) in Colombia. The results are based on 100 posterior trees obtained by continuous phylogeographic inference. The least-cost path was used as the movement model for computing the weight of each environmental variable. Each variable was tested as a factor conducting ("C") or resisting ("R") viral lineages. As described previously (*10*), we report both the median estimate and the 95% HPD interval for regression coefficients and Q values. The Bayes factor (BF) supports are only reported when p(Q > 0) is at least 85% (bold) and only a BF value >20 was considered strong support for significant correlation.

Wave	Environmental factor	Regression Coefficient	Q statistic	p(Q>0)	BF
2	Elevation (C)	8.26e-03 [-7.26e-04 – 1.67e-01]	-1.74e-02 [-4.47e-02 – -1.93e-03]	0	-
	Elevation (R)	2.82e-07 [2.01e-07 – 3.74e-06]	2.88e-03 [-1.18e-02 – 1.52e-02]	0.74	-
	Population density (C)	2.11e-03 [1.30e-03 – 2.68e-02]	2.17e-03 [-8.03e-03 – 1.21e-02]	0.75	-
	Population density (R)	6.61e-06 [3.54e-06 – 8.53e-05]	-5.40e-03 [-2.11e-02 – 2.52e-03]	0.08	-
	Daily temperature(C)	1.83e-04 [9.52e-05 – 2.73e-03]	-3.53e-03 [-9.36e-03 – 3.93e-04]	0.05	-
	Daily temperature(R)	5.35e-07 [3.21e-07 – 2.29e-06]	-4.21e-03 [-4.53e-02 – 1.71e-02]	0.3	-
	Vaccination (C)	-	-	-	-
	Vaccination (R)	-	-	-	-
3	Elevation (C)	1.92e-03 [-5.97e-04 – 4.23e-03]	-5.04e-04 [-2.87e-03 – 5.18e-04]	0.13	-
	Elevation (R)	-5.18e-09 [-4.19e-08 – 3.35e-08]	-1.16e-03 [-3.60e-03 – 1.65e-03]	0.17	-
	Population density (C)	2.25e-04 [-6.11e-05 – 4.02e-04]	-2.50e-04 [-1.14e-03 – 4.31e-04]	0.19	-
	Population density (R)	7.93e-07 [8.94e-08 – 1.38e-06]	2.78e-05 [-8.04e-04 – 1.04e-03]	0.54	-
	Daily temperature(C)	1.91e-05 [-3.38e-06 – 4.27e-05]	-4.09e-04 [-2.93e-03 – 7.56e-04]	0.19	-
	Daily temperature(R)	-8.24e-08 [-1.41e-07 – 1.17e-08]	-2.78e-04 [-3.89e-03 – 2.37e-03]	0.43	-
	Vaccination (C)	9.34 [-1.54 – 11.9]	-1.95e-02 [-2.41e-02 – -8.49e-05]	0	-
	Vaccination (R)	1.23e-10 [3.69e-12 – 1.47e-10]	5.16e-03 [-1.57e-03 – 1.08e-02]	**0.89**	1.9412

Table S6. Rescaling analysis based on the environmental variables detected as potential impactors on the dispersal velocity of SARS-CoV-2 lineages from the locally dispersed epidemic wave 3 in Colombia. Analysis was performed as described previously(*10*) following the expression $v_R = 1 + k * v_0$, where k is the scaling factor and v_0 is the raster for the variable assessed. For regression coefficients and Q values, we report both the median estimate and the 95% HPD interval. The Bayes factor (BF) supports are only reported when p(Q > 0) is at least 85% (bold) and only a BF value >20 was considered strong support for significant correlation.

Environmental factor	*k*	Regression Coefficient	Q statistic	p(Q>0)	BF
Vaccination (R)	10	1.23e-16 [3.69e-18 – 1.47e-16]	5.16e-03 [-1.57e-03 – 1.08e-02]	**0.89**	1.7778
Vaccination (R)	100	1.23e-18 [3.69e-20 – 1.47e-18]	5.16e-03 [-1.57e-03 – 1.08e-02]	**0.89**	1.8571
Vaccination (R)	1000	1.23e-21 [3.69e-23 – 1.47e-21]	5.16e-03 [-1.57e-03 – 1.08e-02]	**0.89**	1.7778

SI References:

1. W. T. Harvey, A. M. Carabelli, B. Jackson, R. K. Gupta, E. C. Thomson, E. M. Harrison, C. Ludden, R. Reeve, A. Rambaut, S. J. Peacock, D. L. Robertson, SARS-CoV-2 variants, spike mutations and immune escape. *Nature Reviews Microbiology* **19**, 409-424 (2021).
2. D. Planas, D. Veyer, A. Baidaliuk, I. Staropoli, F. Guivel-Benhassine, M. M. Rajah, C. Planchais, F. Porrot, N. Robillard, J. Puech, M. Prot, F. Gallais, P. Gantner, A. Velay, J. Le Guen, N. Kassis-Chikhani, D. Edriss, L. Belec, A. Seve, L. Courtellemont, H. Péré, L. Hocqueloux, S. Fafi-Kremer, T. Prazuck, H. Mouquet, T. Bruel, E. Simon-Lorière, F. A. Rey, O. Schwartz, Reduced sensitivity of SARS-CoV-2 variant Delta to antibody neutralization. *Nature* **596**, 276-280 (2021).
3. I. Kimura, Y. Kosugi, J. Wu, J. Zahradnik, D. Yamasoba, E. P. Butlertanaka, Y. L. Tanaka, K. Uriu, Y. Liu, N. Morizako, K. Shirakawa, Y. Kazuma, R. Nomura, Y. Horisawa, K. Tokunaga, T. Ueno, A. Takaori-Kondo, G. Schreiber, H. Arase, C. Motozono, A. Saito, S. Nakagawa, K. Sato, The SARS-CoV-2 Lambda variant exhibits enhanced infectivity and immune resistance. *Cell Reports* **38**, 110218 (2022).
4. F. Fratev, R346K Mutation in the Mu Variant of SARS-CoV-2 Alters the Interactions with Monoclonal Antibodies from Class 2: A Free Energy Perturbation Study. *J Chem Inf Model* **62**, 627-631 (2022).
5. K. Tao, P. L. Tzou, J. Nouhin, R. K. Gupta, T. De Oliveira, S. L. Kosakovsky Pond, D. Fera, R. W. Shafer, The biological and clinical significance of emerging SARS-CoV-2 variants. *Nature Reviews Genetics* **22**, 757-773 (2021).
6. T. S. Tan, M. Toyoda, H. Ode, G. Barabona, H. Hamana, M. Kitamatsu, H. Kishi, C. Motozono, Y. Iwatani, T. Ueno, Dissecting Naturally Arising Amino Acid Substitutions at Position L452 of SARS-CoV-2 Spike. *J Virol* **96**, e0116222 (2022).
7. T.-J. Yang, P.-Y. Yu, Y.-C. Chang, K.-H. Liang, H.-C. Tso, M.-R. Ho, W.-Y. Chen, H.-T. Lin, H.-C. Wu, S.-T. D. Hsu, Effect of SARS-CoV-2 B.1.1.7 mutations on spike protein structure and function. *Nature Structural & Molecular Biology* **28**, 731-739 (2021).
8. A. Escalera, A. S. Gonzalez-Reiche, S. Aslam, I. Mena, M. Laporte, R. L. Pearl, A. Fossati, R. Rathnasinghe, H. Alshammary, A. Van De Guchte, K. Farrugia, Y. Qin, M. Bouhaddou, T. Kehrer, L. Zuliani-Alvarez, D. A. Meekins, V. Balaraman, C. Mcdowell, J. A. Richt, G. Bajic, E. M. Sordillo, M. Dejosez, T. P. Zwaka, N. J. Krogan, V. Simon, R. A. Albrecht, H. Van Bakel, A. García-Sastre, T. Aydillo, Mutations in SARS-CoV-2 variants of concern link to increased spike cleavage and virus transmission. *Cell Host & Microbe* **30**, 373-387.e377 (2022).
9. S. Dellicour, R. Rose, N. R. Faria, P. Lemey, O. G. Pybus, SERAPHIM: studying environmental rasters and phylogenetically informed movements. *Bioinformatics* **32**, 3204-3206 (2016).
10. S. Dellicour, S. Lequime, B. Vrancken, M. S. Gill, P. Bastide, K. Gangavarapu, N. L. Matteson, Y. Tan, L. du Plessis, A. A. Fisher, M. I. Nelson, M. Gilbert, M. A. Suchard, K. G. Andersen, N. D. Grubaugh, O. G. Pybus, P. Lemey, Epidemiological hypothesis testing using a phylogeographic and phylodynamic framework. *Nat Commun* **11**, 5620 (2020).
